# Transcriptional Profiling in Experimental Visceral Leishmaniasis Reveals a Broad Splenic Inflammatory Environment that Conditions Macrophages toward a Disease-Promoting Phenotype

**DOI:** 10.1371/journal.ppat.1006165

**Published:** 2017-01-31

**Authors:** Fanping Kong, Omar A. Saldarriaga, Heidi Spratt, E. Yaneth Osorio, Bruno L. Travi, Bruce A. Luxon, Peter C. Melby

**Affiliations:** 1 Bioinformatics Program, University of Texas Medical Branch, Galveston, Texas, United States of America; 2 Department of Biochemistry and Molecular Biology, University of Texas Medical Branch, Galveston, Texas, United States of America; 3 Department of Internal Medicine, Division of Infectious Diseases, University of Texas Medical Branch, Galveston, Texas, United States of America; 4 Department of Preventive Medicine and Community Health, University of Texas Medical Branch, Galveston, Texas, United States of America; 5 Department of Microbiology and Immunology, University of Texas Medical Branch, Galveston, Texas, United States of America; 6 Center for Tropical Diseases and Institute for Human Infection and Immunity, University of Texas Medical Branch, Galveston, Texas, United States of America; 7 Department of Pathology, University of Texas Medical Branch, Galveston, Texas, United States of America; Queensland Institute of Medical Research, AUSTRALIA

## Abstract

Visceral Leishmaniasis (VL), caused by the intracellular protozoan *Leishmania donovani*, is characterized by relentlessly increasing visceral parasite replication, cachexia, massive splenomegaly, pancytopenia and ultimately death. Progressive disease is considered to be due to impaired effector T cell function and/or failure of macrophages to be activated to kill the intracellular parasite. In previous studies, we used the Syrian hamster (*Mesocricetus auratus*) as a model because it mimics the progressive nature of active human VL. We demonstrated previously that mixed expression of macrophage-activating (IFN-γ) and regulatory (IL-4, IL-10, IL-21) cytokines, parasite-induced expression of macrophage arginase 1 (Arg1), and decreased production of nitric oxide are key immunopathologic factors. Here we examined global changes in gene expression to define the splenic environment and phenotype of splenic macrophages during progressive VL. We used RNA sequencing coupled with *de novo* transcriptome assembly, because the Syrian hamster does not have a fully sequenced and annotated reference genome. Differentially expressed transcripts identified a highly inflammatory spleen environment with abundant expression of type I and type II interferon response genes. However, high IFN-γ expression was ineffective in directing exclusive M1 macrophage polarization, suppressing M2-associated gene expression, and restraining parasite replication and disease. While many IFN-inducible transcripts were upregulated in the infected spleen, fewer were induced in splenic macrophages in VL. Paradoxically, IFN-γ enhanced parasite growth and induced the counter-regulatory molecules Arg1, Ido1 and Irg1 in splenic macrophages. This was mediated, at least in part, through IFN-γ-induced activation of STAT3 and expression of IL-10, which suggests that splenic macrophages in VL are conditioned to respond to macrophage activation signals with a counter-regulatory response that is ineffective and even disease-promoting. Accordingly, inhibition of STAT3 activation led to a reduced parasite load in infected macrophages. Thus, the STAT3 pathway offers a rational target for adjunctive host-directed therapy to interrupt the pathogenesis of VL.

## Author Summary

Visceral leishmaniasis (VL) is a neglected parasitic disease that is caused by the intracellular protozoan *Leishmania donovani*. Patients with this disease suffer from muscle wasting, enlargement of the spleen, reduced blood counts and ultimately will die without treatment. Progressive disease is considered to be due to impaired cellular immunity, with T cell or macrophage dysfunction, or both. We studied the Syrian hamster as an infection model because it mimics the progressive nature of human disease. We examined global changes in gene expression in the spleen and splenic macrophages during experimental VL and identified a highly inflammatory spleen environment with abundant expression of interferon and interferon-response genes that would be expected to control the infection. However, the high level of IFN-γ expression was ineffective in mediating a protective macrophage response, restraining parasite replication and halting progression of disease. We found that IFN-γ itself stimulated parasite growth in splenic macrophages and induced expression of counter-regulatory molecules, which may paradoxically make the host more susceptible. These data give insights into the nature of the immune response that promotes the infection, and identifies potential targets for therapeutic intervention.

## Introduction

Visceral leishmaniasis (VL), caused by the intracellular protozoa *Leishmania donovani* and *L*. *infantum* (syn *L*. *chagasi*), affects nearly a half-million people each year [1]. It occurs in tropical and subtropical regions of the world and is commonly associated with poverty. Infection is initiated when parasites are deposited in the skin by the sand fly vector. In most infected people, the infection is controlled by a type 1 cellular immune response and there are no signs of disease. However, some infected individuals develop a chronic progressive illness characterized by fever, splenomegaly, cachexia, pancytopenia and a relentlessly increasing parasite burden in the spleen, liver and bone marrow. Susceptibility is associated with decreased antigen-induced IFN-γ and IL-12 responses in peripheral blood mononuclear cells [2,3], CD8 T cell exhaustion [4], reduced T cell-mediated macrophage activation and parasite killing [5], and increased IL-10 production [6–8]. In contrast to the *in vitro* finding of decreased antigen-induced IFN-γ, there is a high level of plasma and splenic IFN-γ production [6,9–11] and evidence of antigen-induced IFN-γ production in *ex vivo* whole blood assays [12] in patients with VL. The disconnect between what should be a protective IFN-γ response and the relentless parasite replication and disease progression in VL remains an enigma. *In vitro* models of *L*. *donovani* infection identified several pathways of impaired macrophage function [13], but macrophage function *in vivo* has not been investigated.

*L*. *donovani* infection of Syrian hamsters (*Mesocricetus auratus*) leads to disease that mimics the clinicopathological features of active human VL [14]. We have studied this model to better understand the immunopathogenic mechanisms that lead to progressive VL. We demonstrated that in the spleen of hamster with VL, like in human disease, there is strong expression of IFN-γ that inexplicably does not protect against the relentlessly increasing parasite burden [15,16]. This suggested that splenic macrophages did not effectively respond to classic macrophage activating signals, or responded in a way that was not protective. Indeed, expression of macrophage nitric oxide synthase (NOS2 or iNOS), the primary anti-leishmanial effector mechanism in mice [17], was impaired in macrophages in hamster VL [16,18]. We found evidence for several mechanisms that could account for this, including polarization of macrophages toward an M2-like phenotype with STAT6-dependent dominant arginase expression [19,20], and simultaneous expression of the macrophage suppressive cytokines IL-4 and IL-10 [15,19,21].

To better understand the immunopathogenesis of this disease and macrophage function in the infected tissue environment, we determined global gene expression in infected spleens and splenic macrophages. We used RNA sequencing (RNA-Seq) with *de novo* transcriptome assembly because the hamster genome has not been fully sequenced and/or annotated. Other groups have used this approach to enable transcriptional profiling in non-model organisms [22–26]. RNA-Seq permits cost-effective, simultaneous sequencing at unprecedented scale and speed to quantitatively characterize gene transcription [27]. Analysis of the transcriptional profile in the *L*. *donovani* infected hamster spleen revealed a strikingly proinflammatory environment. There was a remarkable breadth and magnitude of upregulated transcripts related to interferon signaling in the spleen. However, splenic macrophages isolated from hamsters with VL showed fewer differentially expressed transcripts, expressed fewer IFN-response genes, and had a transcriptional profile indicative of a mixed M1- and M2-like activation phenotype. In fact, IFN-γ paradoxically enhanced parasite growth and induced the counter-regulatory molecules Arg1, Ido1 and Irg1 in splenic macrophages. This was mediated, at least in part, through IFN-γ-induced STAT3 activation and expression of IL-10, which suggests that splenic macrophages in VL are conditioned by the chronic inflammatory environment to respond to macrophage activation signals with an exuberant counter-regulatory response that contributes to the progressive infection. The STAT3 pathway offers a rational target for adjunctive host-directed therapy to interrupt the pathogenesis of VL.

## Results and Discussion

### *de novo* assembly of the hamster transcriptome

**Fig 1:**
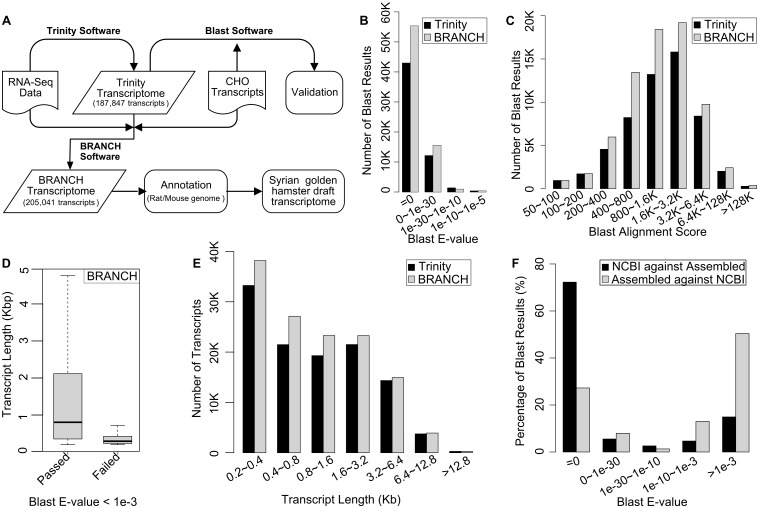
Generation of a Syrian hamster *de novo* assembled transcriptome. **(A)** Summary of the RNA sequencing and transcriptome assembly workflow. **(B)** E-value of BLAST results of transcripts generated by the Trinity and BRANCH software. **(C)** Distribution of BLAST alignment scores generated by the Trinity and BRANCH software. **(D)** Lengths of transcripts generated by BRANCH that passed or failed a BLAST E-value threshold of <1e-3. **(E)** Distribution of number of transcripts generated by the Trinity and BRANCH software according to length of transcript. **(F)** Percent BLAST alignment of the assembled transcriptome with the published NCBI *Mesocricetus auratus* genome, and conversely, the percent BLAST alignment of the NCBI genome with our *de novo* assembled transcriptome.

We evaluated global gene expression in spleen tissue and splenic macrophages in the Syrian (Golden) hamster (*Mesocricetus auratus)* model of progressive VL. *de novo* assembly of a transcriptome was necessary because sequences derived from Chinese Hamster Ovary cells (from its near relative *Crisetulus griseus*) [28], and a draft genome of *Mesocricetus auratus* via genome shotgun sequencing (https://www.ncbi.nlm.nih.gov/bioproject/77669), were incompletely sequenced and/or annotated. To avoid using low quality and artificial sequences, we first performed a quality control analysis of the raw RNA sequencing data. Phred score medians at all bases were ≥30 (i.e., error rate ≤ 0.001) and the majority of the reads had average phred score >37 (S1A and S1B Fig). CG content per read was similar to the theoretical distribution (S1C Fig) and per base N content at each position was <5% (S1D Fig). Control and infected samples generated high quality sequencing reads with a low frequency of sequence artifacts and low quality reads (less than 2%), which were filtered out. We further removed reads that mapped to the *Leishmania donovani* genome (NCBI BioProject PRJEA61817) [29] and assembled a high quality *de novo* transcriptome using Trinity software. Trinity has been proven effective in generating high quality *de novo* transcriptomes with low base-error rates and acceptable accuracy of RNA-Seq reads from non-model organisms [23]. A summary of the workflow is shown in Fig 1A. Trinity produced 187,847 transcripts ranging from 201 to 23,840 nucleotides in length. To validate the assembled results, we compared each transcript against the CHO RefSeq genome (GenBank Assembly ID GCF_000223135.1) by Basic Local Alignment Search Tool (BLAST), which can be exploited to assign a gene ID by identifying homologues in close species [30]. Using the hit with the lowest E-value and the largest alignment score, the largest reported E-value was 1e-5 and 78% of the hits had an E-value equal to 0 (Fig 1B). The majority of the hits returned alignment scores >400 (Fig 1C). These data indicated that the Syrian hamster *de novo* assembled transcriptome was highly homologous to sequences in the CHO-K1 genome, but that it contained more transcript sequences than what is represented or annotated in the CHO RefSeq genome.

We used the BRANCH software to expand the Trinity transcriptome into a more complete transcriptome [22]. With BRANCH, 205,041 transcripts ranging from 201 to 23,840 nucleotides in length were obtained. 64% (131,021) of the BRANCH transcripts had a BLAST E-value <1e-3 when compared to the rat and mouse genomes. The transcripts that passed the E-value cutoff were typically longer and thus more informative than those that failed (Fig 1D). After application of the <1e-3 cutoff to the assembled transcripts, BRANCH produced more transcripts compared to Trinity (Fig 1E). These data indicated that BRANCH improved the Trinity assembly, so we pooled the BRANCH transcripts with an E-value <1e-3 to generate a draft reference transcriptome. Using the alignment software Bowtie2 (v2.1.0), the alignment of the RNA-Seq data to this draft transcriptome had a 92.76 ± 0.68% alignment rate, which was considerably higher than the 58.46 ± 1.38% and 37.77 ± 1.25% obtained when the sequences were aligned to the incomplete NCBI *Mesocricetus auratus* genome (NCBI BioProject PRJNA210213) and CHO RefSeq transcripts. Additionally, we compared our assembled transcriptome with the NCBI *Mesocricetus auratus* genome and found >70% of NCBI transcripts could be found in our *de novo* assembled transcriptome, while <30% of our assembled transcripts were represented (or annotated) in the NCBI genome (Fig 1F). Collectively, these data indicate that the *de novo* assembled and annotated transcriptome is to date the most complete compilation of hamster transcripts available.

### Spleen adherent cells have markers of macrophages

**Fig 2:**
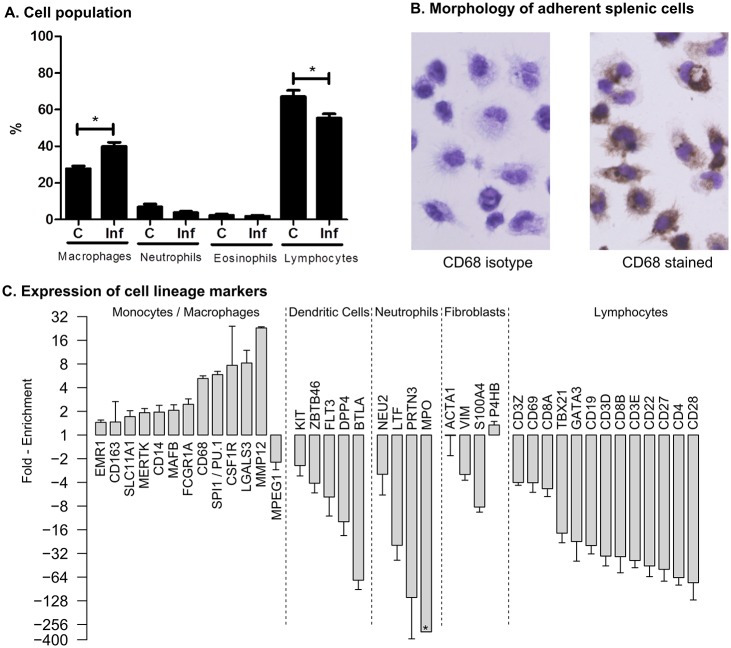
Characterization of a highly enriched macrophage population from the hamster spleen. **(A)** Percent of macrophages, neutrophils, eosinophils and lymphocytes determined by microscopy of Hematoxylin and Eosin stained cytospin preparations of spleen cells from uninfected control (C) and 28-day *L*. *donovani* infected (Inf) hamsters (n = 4 per group). *p<0.05 **(B)** Immunostaining of the enriched splenic macrophage population for intracellular CD68. An isotype-matched antibody was used as a control, and cells were counterstained with Mayer’s hematoxylline. **(C)** Expression of specific cell lineage markers in the enriched splenic macrophage population. Data are shown as the fold-enrichment of sequence counts in the splenic macrophages relative to the total spleen tissue.

Consistent with our previous observations [31], we determined that during the course of experimental VL, there is expansion of splenic macrophages (Fig 2A). We demonstrated previously that this population is parasite-permissive and disease-promoting [16,19,20]. Therefore, their transcriptional profile, in the context of the diseased spleen, was of considerable interest. Antibodies for purification of hamster macrophages are not available, so we used adherence to isolate this population from whole spleen cells from control and infected hamsters. Heavily infected macrophages are less-adherent so may have been underrepresented in the purified population. The purity of this population was demonstrated by their typical macrophage morphology and intracellular expression of the macrophage marker CD68 (Fig 2B). The enrichment of transcripts characteristic of the macrophage lineage, and the absolute or relative absence of transcripts specific to other cell lineages (Fig 2C and S1 Table) confirmed the purity of the isolated macrophages. Note that the transcription factor SPI1 (PU.1), which is involved in the differentiation of macrophages, was also highly enriched in the splenic macrophage population. The enrichment of the fibroblast markers P4HB in the adherent spleen cells is most likely the consequence of expression by inflammatory macrophages [32,33], but we cannot exclude the possible contamination with a small number of fibroblasts. Collectively, these data indicate that the adherent spleen cell population was highly enriched for splenic macrophages.

### Differential gene expression in *L*. *donovani* infected spleens and splenic macrophages

**Fig 3:**
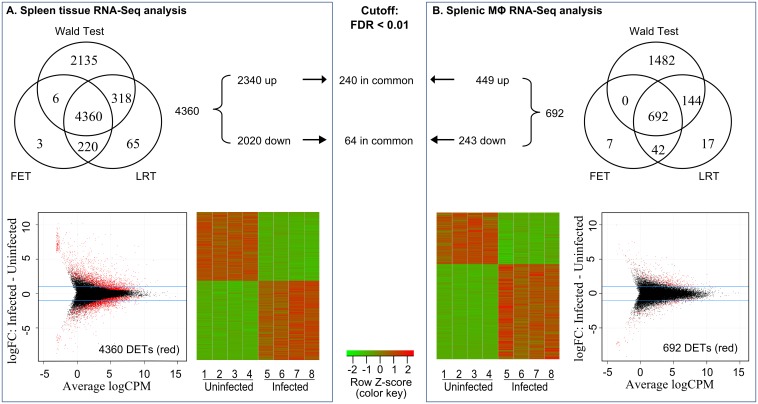
Identification of differentially expressed transcripts in spleen tissue and splenic macrophages in hamsters with VL. The number of differentially expressed transcripts in spleen **(A)** and splenic macrophages **(B)** from hamsters with VL determined by exact tests (FET) and generalized linear models with the likelihood ratio test (LRT) using the BioConductor R package EdgeR, and the Wald test in DESeq. A false discovery rate (FDR) <0.01 was used as the cutoff. Only transcripts with at least 1 count per million in at least 3 out of 4 samples in the control or experimental group were included in the analysis. A transcript was considered differentially expressed only when it was identified by all three different approaches. The lower panels show volcano plots and heat maps of the differentially expressed transcripts (DETs).

The *de novo* assembled annotated transcriptome was used as a draft reference genome for differential gene expression analysis. Multidimensional Scaling (Principal Component Analysis) plots revealed that infected and uninfected samples were appropriately clustered in both spleen and splenic macrophage samples. However, the first dimensional coordinate separated spleen tissue but not splenic macrophage samples (S2A and S2B Fig), which suggests a greater effect of *Leishmania* infection in the whole spleen samples than in the macrophage population. We performed differential expression analysis, using two R BioConductor packages EdgeR and DESeq2 [34–36], between uninfected controls and *Leishmania* infected whole spleen and purified splenic macrophages (both from 28-day infected hamsters). We chose this time point because at this time in the course of infection there is dramatic increase in spleen size, parasite burden and change of splenic macrophages to a more permissive phenotype [20,21,37]. We considered a transcript to have significant differential expression when it was detected by each of the 3 different approaches (see methods). At a False Discovery Rate (FDR) cutoff <0.01 we identified 4,360 differentially expressed transcripts in the spleen samples, which included 2,340 (53.7%) up-regulated and 2,020 (46.3%) down-regulated genes (Fig 3A). At the FDR <0.01 cutoff, splenic macrophages had substantially fewer differentially expressed transcripts (n = 692), which included 449 (64.9%) that were up-regulated and 243 (35.1%) that were down-regulated (Fig 3B). Some differentially expressed transcripts were common to both the spleen and splenic macrophage samples (240 up-regulated and 64 down-regulated) (Fig 3A and 3B), but less <10% of the differentially expressed transcripts in the spleen samples were also differentially expressed in splenic macrophages. The number of differentially expressed transcripts in the spleen tissue and splenic macrophages was decreased to 2778 and 363, respectively, by tightening the FDR to < 0.001 (S2C Fig).

### Molecular pathways are activated or repressed in the spleen and splenic macrophages during VL

**Fig 4:**
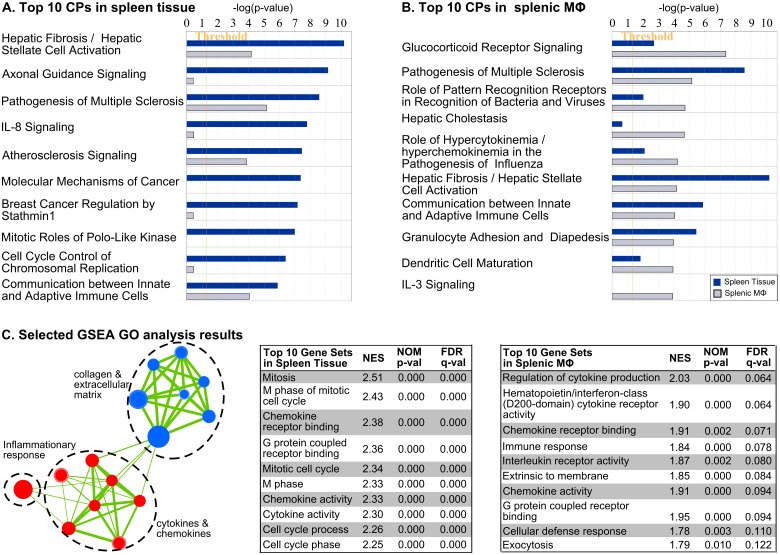
Functional characterization of differentially expressed transcripts in the spleen and splenic macrophages from hamsters with VL. **(A)** Top 10 canonical pathways (CPs) in the spleen (blue bars) compared to splenic macrophages (gray bars). The pathways were generated by loading all transcripts into IPA. **(B)** Top 10 canonical pathways (CPs) in splenic macrophages (gray bars) compared to spleen tissue (blue bars). **(C)** Top 10 gene sets identified in spleen and splenic macrophages determined by Gene Set Enrichment Analysis (GSEA) and Gene Ontology (GO) analysis. The Normalized Enrichment Score (NES), nominal p value, and False Discovery Rate (FDR) are shown for each gene set in the table. A network representation of the inflammatory response, cytokines and chemokines, and collagen and extracellular matrix gene sets is shown.

We identified the molecular pathways significantly altered during infection using the Ingenuity Pathway Analysis (IPA) software package. The top 10 pathways identified as enriched in the 28-day infected spleen and splenic macrophages are shown in Fig 4A and 4B, respectively. A list of the top 50 canonical pathways for both samples can be found in S3A and S3B Fig. A number of the top pathways (Hepatic fibrosis, pathogenesis of multiple sclerosis, atherosclerosis signaling, communication between innate, adaptive immune cells, and the glucocorticoid receptor signaling pathways) identified in whole spleen tissue were also identified in splenic macrophages, supporting the central importance of macrophages in the immunopathogenesis of the splenic infection.

### The splenic environment in experimental VL is highly proinflammatory

**Fig 5:**
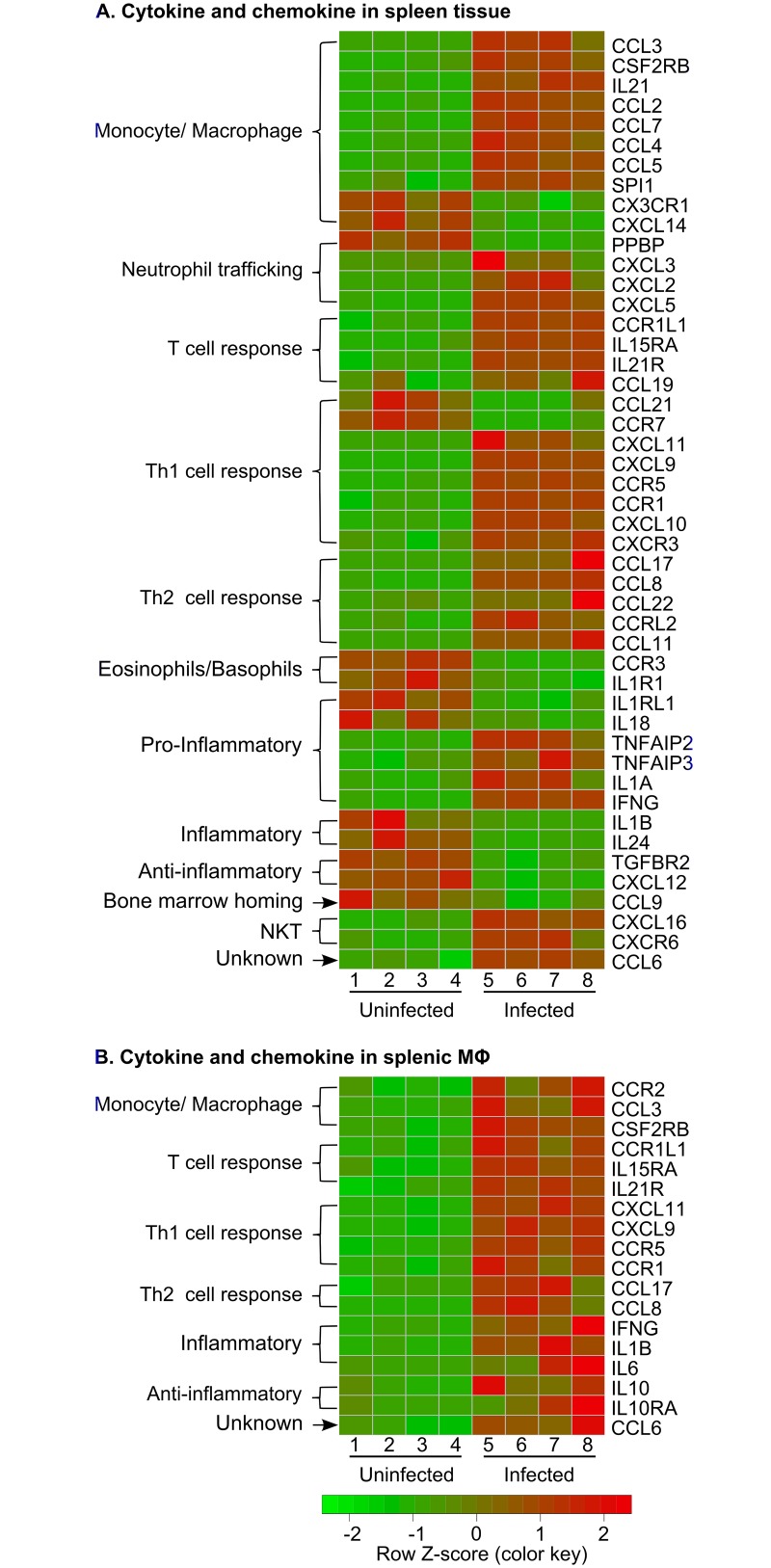
Differential expression of cytokine and chemokine transcripts in spleens and splenic macrophages of hamsters with VL. Heat maps showing the differential expression of selected transcripts in spleen tissue **(A)** and splenic macrophages **(B)** in uninfected (left side) and 28-day *L*. *donovani* infected (right side) hamsters (n = 4 per group). Transcripts down-regulated during infection are shown in green and upregulated transcripts are in red. The source or general function of the cytokine or chemokine is annotated to the left.

A common characteristic shared by several of the enriched pathways in the infected spleen and splenic macrophages was the upregulation of inflammatory cytokines, chemokines and their receptors. The significance of these molecules was also confirmed by Gene Set Enrichment Analysis (GSEA), which revealed that at least 4 out of the top 10 gene sets enriched in spleen and splenic macrophages, were associated with production, receptor activity and signaling of cytokines and chemokines. The list of these gene clusters and their interaction networks are shown in Figs 4C and S3C. Differentially expressed cytokines, chemokines, and their receptors identified the broad inflammatory nature of the spleen during VL and suggested involvement of multiple leukocyte populations (Fig 5; S2 Table). In the spleen, transcription factors that drive inflammation, including those involved in interferon and cytokine responses (STAT1, STAT2, STAT3, IRF1, IRF7, XBP1, LITAF) and MHC expression (XBP1, NLRC5), were both transcriptionally upregulated and predicted to be activated during infection (S3 Table). Additionally, transcription factors involved in regulation of the inflammatory response, including the NF-kB complex (RELA, RELB), TBX21, NFATC2 (T cell activation), STAT4, IRF3/5, IFI16, HMGB1, BCL10 (NF-kB activator), CBP/P300, and DDIT3 (caspase activation, cytokine expression), were predicted to be activated in the infected spleen tissue despite some not being differentially expressed (S3 Table). The predicted activation of lipopolysaccharide-induced TNF factor (LTIF) [38] and increased expression of TLR4 in the splenic macrophages suggested that circulating LPS may might be a contributor to the proinflammatory nature of the spleen in VL. Elevated circulating endotoxin levels resulting from increased intestinal permeability and bacterial translocation were observed during human VL [39]. In hamsters with VL we found serum endotoxin levels to be highly variable without a significant increase in mean levels in infected vs. uninfected hamsters (15.9 vs. 8.4, *p* = 0.13; S4 Fig). However, the proportion of infected hamsters with a high circulating endotoxin level (>30 EU/mL) was significantly greater in the infected compared to uninfected animals (40% vs. 5%; *p* = 0.027 by Fisher Exact Test).

Fewer cytokine and chemokine mRNAs were differentially expressed in splenic macrophages compared to whole spleen tissue, but all of the differentially expressed cytokine/chemokine transcripts in splenic macrophages were upregulated. Notably, proinflammatory macrophage-activating cytokines (IFN-γ, IL-1β) were upregulated, as were receptors that would be responsive to inflammatory signals (e.g. toll-like receptor-4 [TLR4], IL-15Rα, CSF2Rβ/IL-5Rβ [common subunit of the IL-3, IL-5, and GM-CSF receptors] and IL-21R) (Fig 5B; S2 Table). These findings distinctly contrast with data from *in vitro* infected mouse [40,41] and human [42] macrophages, which indicated that *Leishmania* infection had a broadly silent or suppressive rather than activating effect on macrophage inflammatory gene expression. Thus, our data suggest that the inflammatory signals generated in the infected spleen environment, which would be absent from *in vitro* infected macrophages, have considerable influence on the activation status of splenic macrophages.

### Diverse expression of myeloid cell chemokines in the spleen

The expansion of myeloid cells in the spleen in VL may result from recruitment from the bone marrow, extramedullary hematopoiesis from *in situ* precursors [43], and/or local proliferation of resident macrophages. Chemokines that act to recruit monocytes/macrophages (CCL2, CCL3, CCL4, CCL5, CCL6 and CCL7) were highly expressed in the spleen during VL. The chemokine receptors CCR1, CCR2, and CCR5 were also increased on splenic macrophages (Fig 5; S2 Table). Spleens from mice infected with *Leishmania chagasi* had sustained expression of CCL2, which was associated with the influx of macrophages that enhanced the infection [44]. Transcription factors that regulate myelopoiesis (Egr2, SPI1, IRF8 and AP1) [45,46] were enriched or predicted to be activated in the splenic macrophage population (S1 and S3 Tables). Thus, local generation may also contribute to the accumulation of myeloid cells in the spleen. We have found that splenic macrophages are highly proliferative during active VL (Osorio, Melby, manuscript in preparation). Expression of neutrophil (CXCL2, CXCL3, CXCL5, and CCL3) and eosinophil (CCL11 and its receptor CCR3) chemoattractants was also increased significantly in the infected spleen, but we found no increase in these cells by morphological analysis of splenocytes (Fig 2A). Better markers for hamster neutrophils are needed to exclude their accumulation in the spleen. It is also possible that the neutropenia commonly found in VL limits their accumulation in the spleen.

### Splenic macrophages in experimental VL demonstrate a mixed polarization/activation phenotype

**Fig 6:**
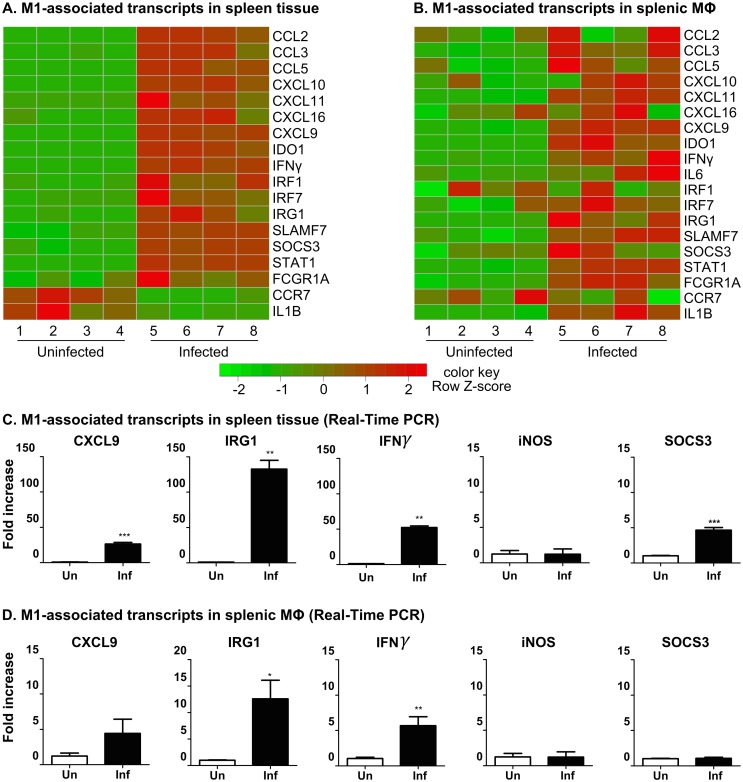
Expression of M1-associated transcripts in spleen and splenic macrophages in VL. Heat maps showing the differential expression of selected M1-associated transcripts in spleen tissue **(A)** and splenic macrophages **(B)** in uninfected (left side) and 28-day *L*. *donovani* infected (right side) hamsters (n = 4 per group). Transcripts down-regulated during infection are shown in green and upregulated transcripts are in red. The expression of selected M1-associated transcripts were confirmed by real-time RT-PCR in spleen tissue **(C)** and splenic macrophages **(D)** from hamsters with VL (n = 6–8 per group). Data are shown as the mean and SEM of the fold-change relative to the uninfected group. *p<0.05; **p<0.01; ***p<0.001.

**Fig 7:**
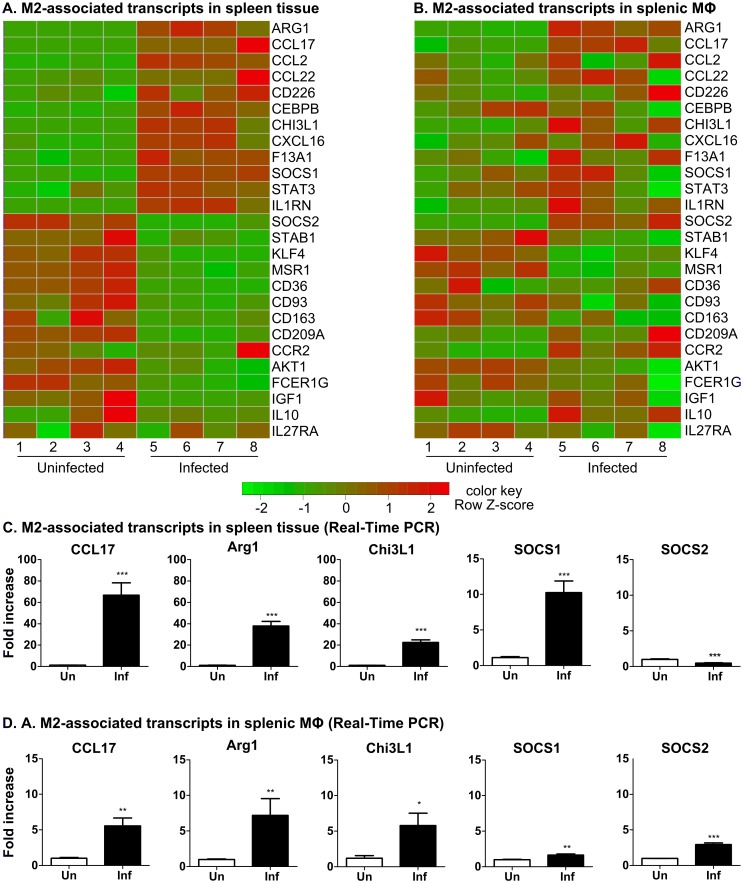
Expression of M2-associated transcripts in spleen and splenic macrophages in VL. Heat maps showing the differential expression of selected M2-associated transcripts in spleen tissue **(A)** and splenic macrophages **(B)** in uninfected (left side) and 28-day *L*. *donovani* infected (right side) hamsters (n = 4 per group). Transcripts down-regulated during infection are shown in green and upregulated transcripts are in red. The expression of selected M2-associated transcripts was confirmed by real-time PCR in spleen tissue **(C)** and splenic macrophages **(D)** from hamsters with VL (n = 6–8 per group). Data are shown as the mean and SEM of the fold-change relative to the uninfected group. *p<0.05; **p<0.01; ***p<0.001.

Macrophages exhibit considerable plasticity in their activation state, which depends on cues received from the local environment [47,48]. The macrophage activation phenotype, and the signals that drive it, is critically important in VL because macrophages have the dual role of mediating intracellular parasite killing and controlling tissue damage and repair [49]. At the extremes of the polarization spectrum, M1 macrophages are important for the clearance of intracellular pathogens including *Leishmania* [50], while M2 macrophages are protective against helminths and have anti-inflammatory and tissue repair functions [49,51]. However, accumulating evidence indicates that in tissue inflammation and infection [52,53], the polarization of macrophages does not always fit neatly within the dichotomous M1–M2 classification system [48]. We evaluated the expression of genes known to be associated with macrophage activation/polarization (see S4 Table for a full list of references). Splenic macrophages from hamsters with VL had a significantly increased expression of genes characteristic of both M1 (CXCL9, CXCL11, IL1B, IL6, FCGR1A, IDO, IRG1, IFNγ, STAT1, CCL3, CCL5) (Fig 6, S4 Table) and M2 (Fig 7, S4 Table) polarization. The M1-associated genes showed a more consistent pattern of upregulation in the infected vs. uninfected groups compared to the M2-associated genes, perhaps suggesting a bias toward the M1 phenotype (compare Figs 6 and 7). Some M1-associated transcripts (OASL, IRF1, IRF7) were upregulated in the spleen tissue but not splenic macrophages, suggesting that other cell populations, possibly fibroblasts [37], may have an immunoregulatory role. Other M1-associated markers (NOS2, CXCL13, IFNGR, CD86, CCR7, CD80, CD68, IL7R, HRH1, BCL2A1, SPHK1, PFKFB3, PSMA2, ATF3) were not upregulated in either spleen tissue or splenic macrophages (data accessible in NCBI's Gene Expression Omnibus [54] through GEO Series accession number GSE91187; http://www.ncbi.nlm.nih.gov/geo/query/acc.cgi?acc=GSE91187). The upregulation of IL-1β, IFN-γ and IL-6 in splenic macrophages (Fig 6; S2 Table) suggests an additional inflammatory effect on the macrophages through paracrine or autocrine activation.

The M2 program of macrophage polarization was classically described as driven by IL-4- or IL-13-induced STAT6 activation [55]. However, there is a spectrum of macrophage phenotypes induced by anti-inflammatory signals such as IL-10, TGF-β, glucocorticoids, and immune complexes [48,52], which overlap with the IL-4/IL-13-polarized phenotype. In hamsters with VL, the splenic macrophages showed significantly increased expression of some M2-associated transcripts, including Arg1, IL-10, SOCS2, CCL17, Chi3L1 (Fig 7, S4 Table). We demonstrated previously that parasite-induced arginase-1 expression in macrophages in VL was dependent on STAT6 activation and amplified by IL-4 and growth factor receptor signaling [19,20]. Increased expression of IL-10 and IL-10R in splenic macrophages (Fig 5; S2 Table) also contributed to arginase expression [20]. IL-10 is also likely to dampen the effects of the proinflammatory cytokines and promote infection through suppression of T cell or macrophage effector function [56,57]. In contrast, other transcripts considered to be markers for M2 macrophages (F13A1, CCL11, FN1, CCL2, CCL22, PPARγ) were not upregulated in splenic macrophages (S4 Table). The M2-associated marker MSR1 (Fig 7, S4 Table) was down-regulated. Furthermore, splenic macrophages did not display upregulation of angiogenic factors (VEGFA, EPHB1/4, DLL4, LYVE1, ANGPT1, NRP1), which are characteristic of M2 activation. CCL2, which was highly upregulated in the spleen in VL, was demonstrated to drive the accumulation of tumor-associated macrophages and together with IL-6 (also found to be upregulated in splenic macrophages) promoted polarization and survival of M2-like macrophages [58].

**Fig 8:**
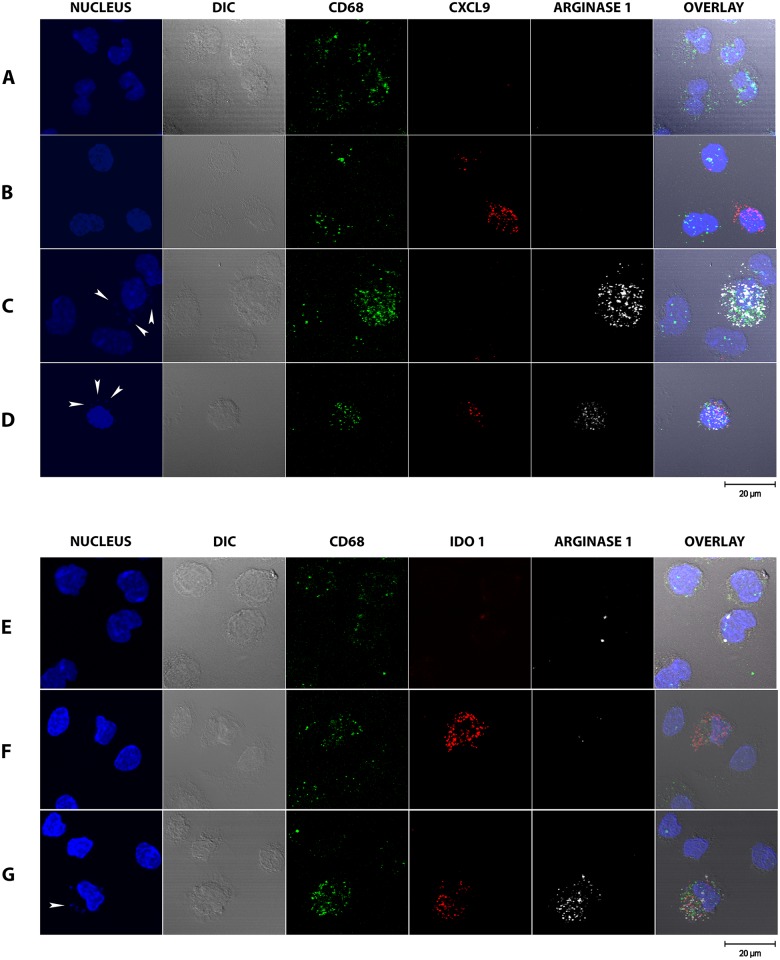
Single-cell expression of Cxcl9, Arg1, and Ido1 RNAs in splenic macrophages from uninfected and *L*. *donovani* infected hamsters. The single-cell expression of M1-associated (Ido1 and Cxcl9) and M2-associated (Arg1) mRNA transcripts and CD68 in splenic macrophages from uninfected (row A) and *L*. *donovani* infected hamsters (rows B, C, and D) was determined by *in situ* amplification and fluorescence hybridization. To determine co-expression, cells were hybridized with fluorescent probes specific to CD68, CXCL9, and Arginase 1 (upper panel) or CD68, IDO1, and Arginase 1 (lower panel) and imaged using confocal microscopy. Nuclei were detected by DAPI staining and cellular morphology by Differential interference contrast (DIC) microscopy. In some cases, parasite nuclei could be seen adjacent to macrophage nuclei (indicated by white arrowheads in the DAPI stained cells). The percent of single-positive and double-positive cells was calculated by counting 100 CD68-positive cells. Uninfected cells (row A) were negative for CXCL9, IDO1, and Arginase 1.

To determine if the coexistent expression of M1- and M2-associated transcripts occurred within the same macrophages or within a mixed population of macrophages, we detected M1-associated (Ido1 and Cxcl9) and M2-associated (Arg1) mRNA transcripts in CD68^+^ splenic macrophages by *in situ* amplification and fluorescence hybridization [59]. We found that the spleen of hamsters with VL contained macrophages that were single-positive for either the M1 or M2 marker, and double-positive for both (Fig 8). Thus, splenic macrophages in VL have a diverse phenotype with evidence of M1, M2 and mixed polarization. Co-expression of M1 and M2 associated genes has also been shown in peritoneal macrophages elicited with *Toxoplama gondii* [52], adipose tissue macrophages [60], GM-CSF knockout mice [61] and monocytes infected with human cytomegalovirus [53]. A mixed phenotype of macrophages could also be present during the transition from acute to chronic stages of infection [62].

### Regulators of splenic macrophage polarization in VL

**Fig 9:**
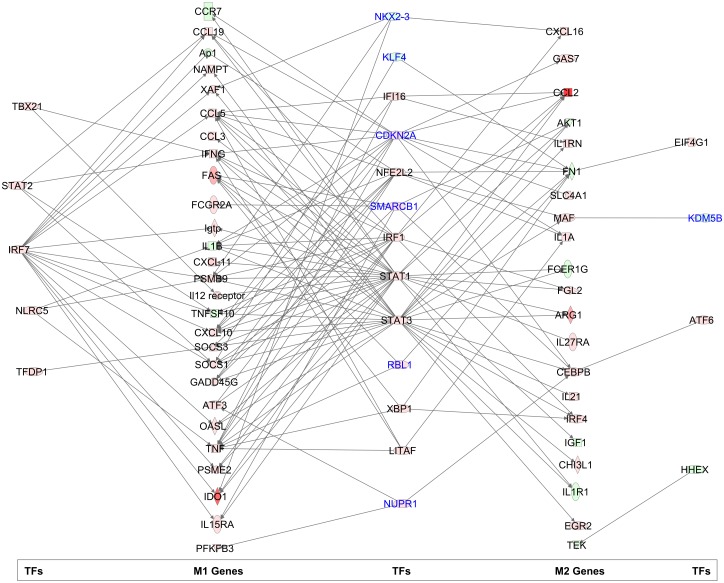
Predicted transcription factor regulation of M1- and M2-associated gene expression in splenic macrophages from hamsters with VL. The set of differentially expressed genes in splenic macrophages was loaded into IPA software to predict the transcription factors likely to be activated. Shown is the overlapping network analysis of these transcription factors and the differentially expressed transcripts.

To better define the regulation of the splenic macrophage transcriptional response we examined differential expression of mRNAs of M1/M2-relevant specific transcription factors. We also loaded the set of differentially expressed genes into IPA software to predict the transcription factors likely to be activated. mRNAs for transcription factors associated with M1 (IRF1, IRF7, STAT1, STAT5A/B) and M2 (IRF4, CEBPβ, STAT3) were upregulated, and except for CEBPβ, were predicted to be activated (S3 Table). The M2-associated transcription factor KLF4 was downregulated and predicted to be inhibited in the spleen (S3 Table). In splenic macrophages, STAT1 mRNA was significantly upregulated and this transcription factor was predicted to be activated, while other M1- (RELA, IRF3, IRF7, JUNB/AP1, NFE2L2/NRF2, IFI16) and M2-associated transcription factors (CEBPα,β; NFKB1/P50) were predicted to be activated (based on downstream gene targets) without increase in their mRNA expression (S3 Table). Overlapping network analysis suggests IRF7 as a primary regulator of M1-associated gene expression, and STAT1, IRF1 and STAT3 as dual regulators of both M1 and M2 gene expression in VL (Fig 9). IRF7 was shown previously to mediate acquired resistance in the liver of *L*. *donovani* infected mice via expression of IFN-γ and iNOS [63]. STAT6, which we showed was activated (phosphorylated) in the spleen in VL and was critically important for splenic arg1 expression [20,37], was neither upregulated at the transcript level or predicted to be activated. This suggests that the effect of STAT6 activation is limited to a relatively small set of genes that do not reach the threshold for detection of pathway activation in the context of global gene expression. Consistent with our previous finding [20], activation of STAT3 was predicted. Its activation may be driven by IL-21, IL-6 and/or IL-10, which were upregulated in the spleen and splenic macrophages. STAT3 may coordinately regulate with STAT6 the expression of M2-associated genes, including ARG1 [20]. STAT3 also has a key role in the expansion and suppressive activity of myeloid derived suppressor cells (see further discussion below) [64]. IRF4, a transcription factor that contributes to M2 polarization [65], showed upregulated mRNA but was not predicted to be activated.

### Interferons and interferon-response signature in VL

**Fig 10:**
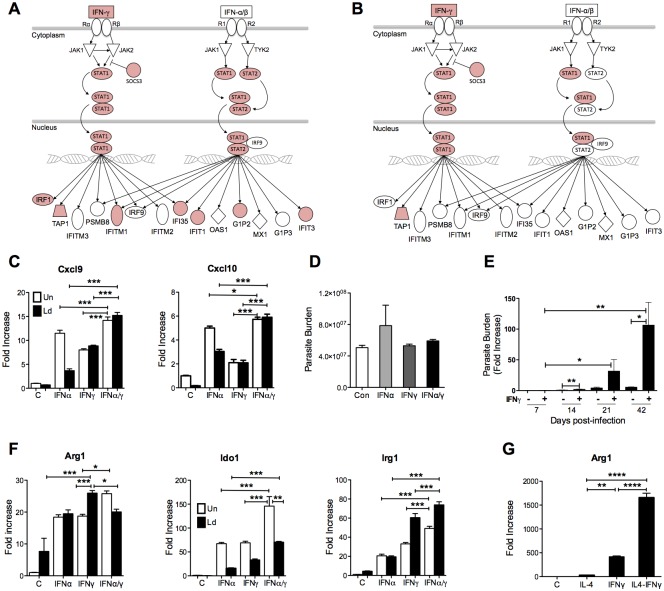
IFN-γ signaling leads to altered gene expression and increased parasite load in infected splenic macrophages. Differentially expressed transcripts from spleen tissue **(A)** and splenic macrophages **(B)** were loaded into IPA and the canonical IFN-γ signaling pathway generated. Transcripts upregulated in infection are shaded in red. **(C)** Expression of Cxcl9 and Cxcl10 in bone marrow derived macrophages (BMDMs) that were uninfected (Un) or infected *in vitro* with *L*. *donovani* (Ld), and left unstimulated (C) or stimulated with IFN-α, IFN-γ, or a combination of both (IFNα/γ) for 24 hrs. Data are shown as the mean and SEM of the fold-change relative to the uninfected, unstimulated group. **(D)** Parasite burden in bone marrow derived macrophages infected *in vitro* with *L*. *donovani* and left unstimulated (Con) or stimulated by IFN-α, IFN-γ, or a combination of both (IFNα/γ) for 24 hrs. **(E)** Relative parasite burden in splenic macrophages isolated from hamsters at 7, 14, 21, and 42 days after *L*. *donovani* infection, cultured and stimulated *ex vivo* for 24 hrs with hamster IFNγ (+) or mock supernatant (-). Parasite load was determined by expression of Leishmania 18S gene and fold-increase calculated against uninfected cells. **(F)** Expression of Arg1, Ido1, and Irg1 in uninfected (Un) and infected (Ld) BMDMs treated for 24 hrs with recombinant mouse IFN-α, recombinant hamster IFN-γ, or a combination of both (IFNα/γ). **(G)** Expression of Arg1 in uninfected BMDMs treated for 24 hrs with recombinant hamster IL-4, IFN-γ, or a combination of both (IL4-IFNγ). *p<0.05; **p<0.01; ***p<0.001.

Consistent with previous studies in this experimental model [15,16,20,21] and human VL [6,9–11], we found IFN-γ was highly upregulated in the spleens of infected hamsters (>50-fold increase). We also found significantly increased splenic expression of >100 known IFN-responsive genes (Figs 10A and S5, S5 Table). These included members of the IFI gene family, interferon-stimulated genes (ISG), guanylate binding proteins, interferon response factors, and antiviral effectors. The repertoire of interferon response genes (compared to a manually curated reference set) was significantly more extensive in the spleen tissue compared to splenic macrophages (99/148 [67%] vs. 52/148 [35%]; *p* = 0.0001 by Fisher Exact Test; S5 Table). Ingenuity Pathway Analysis confirmed that there were fewer IFN-response genes upregulated in splenic macrophages relative to the whole spleen (Fig 10A and 10B). Collectively, these data suggest impaired or altered responsiveness of splenic macrophages to interferons in VL relative to other spleen cell populations. Although the production of IFN-γ is considered to be restricted to T cells and NK cells, we also found increased IFN-γ expression in splenic macrophages from hamsters with VL (11-fold increase) (Fig 6, S4A Table). Other studies demonstrated that murine macrophages expressed IFN-γ in response to LPS, IFNγ, *M*. *tuberculosis*, and *Streptococcus pyogenes* [66–69].

In the environment of the chronically infected spleen, the high level of IFN-γ expression is ineffective in mediating exclusive M1 macrophage polarization, suppressing M2-associated gene expression, and restraining parasite replication and progressive disease. The generation of nitric oxide via macrophage inducible nitric oxide synthase (NOS2) is a key anti-leishmanial effector mechanism in mice [17]. Consistent with our previous observations [16,18], NOS2 was notably absent from the upregulated M1-associated repertoire of genes. NOS2 is a target of IFN-γ via the action of STAT1 and IRF1. These transcription factors were predicted to be activated, so the absence of NOS2 expression suggests its suppression by other regulatory mechanisms. Specific sequences in the hamster NOS2 promoter that render it less responsive to IFN-γ-mediated transactivation (also found in the human NOS2 promoter) [18] may contribute to this. There is also a large body of evidence that supports NOS2 suppression by anti-inflammatory cytokines such as IL-10 (see below).

Evidence for a role of type 1 interferons in *Leishmania* infection is ambiguous. Early type 1 IFN production is protective in murine *L*. *major* infection, probably via promoting or shaping the adaptive Th1 response [70,71]. However, IFN-β impaired parasite killing in human macrophages [72]. Furthermore, sustained pathogen-induced type 1 interferon signaling can promote infection with intracellular bacteria [73–76]. Since many of the upregulated IFN-response genes in VL are induced by type 1 interferons (either uniquely or in common with IFN-γ), we reasoned that the apparent unresponsiveness of infected macrophages to IFN-γ could be due to antagonistic crosstalk with type 1 IFN signaling (reviewed in [77]). Pathway analysis revealed evidence of activation of the type 1 interferon signaling pathway with upregulation of numerous type 1 IFN-responsive genes in the spleen (Figs 10A and S5; S5 Table). Surprisingly, we did not find increased expression of type 1 or type 3 interferon transcripts in the spleen of infected animals in the RNA-Seq dataset (data accessible in NCBI's Gene Expression Omnibus [54] through GEO Series accession number GSE91187; http://www.ncbi.nlm.nih.gov/geo/query/acc.cgi?acc=GSE91187). Since there is considerable heterogeneity in type 1 and type 3 interferons among different animal species, it is possible that some hamster IFN sequences were missed because they did not have sufficient homology to mouse, rat, or human sequences to meet the threshold of identification by BLAST. Alternatively, activation of the type 1 IFN pathway independent of type 1 IFNs, such as may occur via TLR-mediated activation of IRF3 or IRF7 [78,79] (which were predicted to be activated), may contribute to the broad type 1 IFN-response signature.

To evaluate the functional significance of IFN signaling we exposed hamster macrophages to IFN-α, IFN-γ or both. We found that both were effective in inducing the classic IFN-response genes, CXCL9 and CXCL10 (Fig 10C). Pre-exposure to IFN-α did not blunt the IFN-γ-induced transcriptional response but amplified the expression of IFN-response genes (Fig 10C). This indicates there was no IFN-α-induced inhibitory crosstalk. Pre-exposure of bone marrow derived macrophages to IFN-α, like IFN-γ [16], did not lead to parasite killing (Fig 10D). Strikingly, however, splenic macrophages isolated from hamsters with VL showed a dramatic increase in parasite burden when exposed to IFN-γ (Fig 10E). This pathological effect increased over the course of the infection. Collectively, these data indicate that type 1 interferons have neither a direct pathologic or protective role, but IFN-γ has a paradoxical disease promoting effect on macrophages conditioned by the inflammatory environment of the spleen or the high intracellular parasite load in VL.

We found evidence that IFN-γ induces immunomodulatory proteins that are likely to render the macrophage more permissive to parasite replication. Our previous work demonstrated a pathological role for macrophage Arg1 in VL [19,20]. Arg1 is a prototypic IL-4-induced M2 marker in most models (as discussed above). The pathological expression of Arg1 in *L*. *major* infection in mice is driven by a dominant Th2 response [80–82]. In contrast, our data indicate that expression of Arg1 is not part of a conventional Th2-driven M2 macrophage phenotype, but occurs within the highly proinflammatory, IFN-dominated environment of the spleen. We show here for the first time that both IFN-α and IFN-γ are potent inducers of Arg1 in uninfected and infected macrophages (Fig 10F). Furthermore, IL-4 and IFN-γ, both of which are expressed in the spleen in VL, were synergistic in their induction of Arg1 expression (Fig 10G). Since Arg1 expression impairs macrophage and T cell effector function (the latter through depletion of L-arginine), this may be part of the mechanism through which IFN-γ is paradoxically disease-promoting.

The IFN-γ-inducible gene indoleamine 2,3-deoxygenase (IDO-1), which mediates the catalytic degradation of L-tryptophan [83], was highly upregulated in the whole spleen (369-fold) and splenic macrophages (40-fold) of hamsters with VL (Fig 6, S4A Table). IDO-1 was induced in macrophages by IFN-γ and/or IFN-α, and this was amplified by infection with *L*. *donovani* (Fig 10F). Previous studies demonstrated that Ido1 can suppress adaptive immunity through generation of T cell tolerance [83], suppression of the T cell stimulatory capacity of dendritic cells [84], generation of regulatory T cells [85], and polarization of macrophages into an M2-like phenotype [86]. Ido1 has been proposed as a biomarker of active VL in humans [87] and a pathological determinant in experimental *L*. *major* infection [84,88]. Its role in the pathogenesis of VL remains to be determined.

Another IFN-γ-induced transcript, immunoresponsive gene 1 (Irg1) [89] was significantly up regulated in the infected spleen (365-fold) and splenic macrophages (7.8-fold) compared to uninfected controls (Fig 6, S4 Table). Irg1 expression was increased in macrophages following IFN-γ and/or IFN-α exposure, and this was amplified by infection (Fig 10F). Recently, IRG1 was found to suppress macrophage activation via expression of the negative regulator A20 [90]. Thus, the high level of IFN-γ expression in VL may be paradoxically counterproductive by promoting the development of macrophage and/or T cell phenotypes that favor parasite growth and survival. This possibility needs further research.

### Splenic expression of cytokine inhibitors of macrophage effector function in VL

Concomitant with the IFN-γ expression, several anti-inflammatory cytokines that favor parasite growth and survival were expressed in the spleen and/or splenic macrophages. IL-10 and IL-10R were upregulated in splenic macrophages. IL-10 has emerged as a key cytokine that drives VL pathogenesis [6,12] through suppression of effector T cell responses and possibly macrophage function. The binding of immune complexes (known to be abundant in active VL [91]) to Fc-gamma receptors (upregulated in splenic macrophages) can also polarize macrophages to a regulatory IL-10-producing phenotype [92,93]. The pleiotropic cytokines IL-21 and IL-27 can induce the production of IL-10 through the activation of STAT3 [94,95]. Co-expression of splenic IL-21, IL-27 and IL-10, and IL-21-mediated induction of IL-10, was shown in patients with VL [96]. In our model, Il27 mRNA (IL-27p38 subunit) was upregulated 2.7-fold (FDR = 0.045) in the spleen in VL. However, the other subunit of the heterodimer, Eib3, was downregulated (2.0-fold in the spleen and 2.7-fold in splenic macrophages; FDR<0.001 for both). IL-21 and its receptor were upregulated in the spleen, and IL-21R was upregulated in splenic macrophages. Besides its induction of IL-10, IL-21 receptor signaling may also promote infection via M2 polarization of macrophages [97], development of Th2 responses [98], and suppression of dendritic cell activation and maturation [99]. It could also have a role in expansion of splenic hematopoietic progenitor cells [100] and B cells, both of which are associated with an increase in parasite burden [101]. Splenic B cells in turn may promote infection by production of IL-10 and suppression of protective Th1 cell responses [102–104].

Inflammatory cues in a number of infections and cancers lead to accumulation of a heterogeneous population of immature myeloid-derived suppressor cells (MDSC) that have profound anti-inflammatory and immunosuppressive effects [105]. These cells accumulate in the blood, bone marrow, and secondary lymphoid organs and may arise within either the granulocytic or monocytic MDSC lineages [106]. MDSCs are induced by pro-inflammatory cytokines (TNF, IL-6, IL-1), such as we found in the inflammatory environment of the spleen in VL. MDSCs promote immune suppression primarily by dampening T cell responses [105,107], but may also provide a permissive environment for replication of intracellular pathogens. Arg1 and Ido1, which were highly upregulated in splenic macrophages in VL, are common mediators of the immunosuppression conferred by MDSC [105,108]. The high expression of CCL8 in the spleen in VL may also contribute to the expansion of immature myeloid cells having a regulatory phenotype [109]. While tools to detect surface markers for MDSCs in hamsters are not available, our data suggests that splenic MDSCs may have a role in disease progression in VL.

### The parasite-promoting effect of IFN-γ is mediated through STAT3 activation

**Fig 11:**
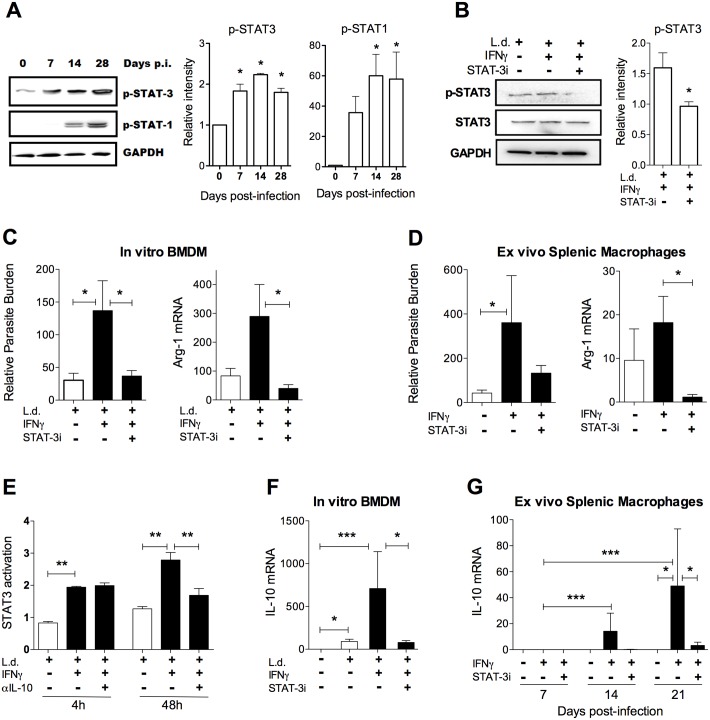
IFN-γ-mediated counter-regulatory response and increased parasite load in macrophages is dependent on STAT3 activation. **(A)** Representative immunoblots showing phosphorylation of STAT1 and STAT3 in splenic macrophages from uninfected (day 0) and *L*. *donovani* infected (day 7, 14 and 28) hamsters. The relative band intensities are graphed from data from 3 experiments with samples pooled from 4 hamsters per time point. (**B)** Representative immunoblot of phospho-STAT3 (p-STAT3) expression in infected splenic macrophages after 20 min of IFN-γ exposure with and without treatment with 100 μM STAT3 inhibitor (STAT-3i) before IFN-γ stimulation. The relative intensities of the p-STAT3 bands are graphed, representative of 3 experiments. **(C)** Relative parasite burden (left panel) and arginase-1 (Arg-1) expression (right panel) in hamster BMDM infected *in vitro* with *L*. *donovani* and treated or not for 24 hrs with IFN-γ, with or without pre-treatment with the STAT3 inhibitor (STAT-3i). Fold change compared to mock treated macrophages. **(D)** Relative parasite burden (left panel) and arginase-1 (Arg-1) expression (right panel) in splenic macrophages from *L*. *donovani* infected hamsters (21 days p.i.), cultured and stimulated *ex vivo* for 24h with or without IFN-γ, with or without pre-treatment with the STAT3 inhibitor (STAT3i). Fold change compared to mock treated macrophages. (**E)** STAT3 Luciferase reporter assay in BHK cells transduced with STAT3 lentiviral reporter (Cignal Lenti, Qiagen) exposed or not for 4h or 48h to *L*. *donovani* with or without IFN-γ, with or without or pre-treatment with 2 μg/mL of anti-mouse/rat IL-10 neutralizing antibody (AF519, R&D). **(F)** Interleukin-10 (IL-10) mRNA expression in hamster BMDM uninfected or infected *in vitro* with *L*. *donovani* and stimulated or not for 24 hours with IFN-γ, with or without pre-treatment with the STAT3i. Fold change compared to mock treated macrophages. (**G)** Interleukin-10 (IL-10) expression in splenic macrophages from hamsters isolated 7, 14, or 21 days after infection with *L*. *donovani*, and cultured and stimulated *ex vivo* with IFN-γ for 24h, with or without pre-treatment with the STAT3 inhibitor (STAT3i). Fold change compared to mock treated macrophages. *p<0.05, **p<0.01, ***p<0.001.

We hypothesized that IFN-γ may increase parasite load in splenic macrophages through the STAT3 pathway, which was predicted by IPA analysis of the RNA-seq dataset to be activated during VL (S3 Table; Fig 9). STAT3 phosphorylation was significantly increased in splenic macrophages from hamsters with VL as early as 7 days post-infection and was sustained throughout the course of infection (Fig 11A). This preceded the activation of STAT1, which was increased at day 14 and 28 post-infection. We used a chemical inhibitor to block STAT3 phosphorylation in infected splenic macrophages exposed to IFN-γ (Fig 11B). Inhibition of STAT3 activation abrogated the IFN-γ-induced increase in parasite load and ARG1 expression in *in vitro* infected bone marrow macrophages (Fig 11C) and *ex vivo* cultured splenic macrophages from hamsters with VL (Fig 11D). We reasoned that IFN-γ could mediate the disease promoting effect through direct STAT3 activation, as has been demonstrated [110,111], or through an indirect pathway involving STAT3-activating counter-regulatory cytokines, such as IL-10. Using a STAT3 reporter assay, we found that exposure to *L*. *donovani* and IFN-γ induced STAT3 activation as early as 4 hrs after exposure (Fig 11E). This early effect would not be dependent on *de novo* synthesis of a secondary protein mediator, and was not affected by IL-10 neutralization (Fig 11E). However, at 48 hrs after exposure the increased IFN-γ-induced STAT3 activation was reduced by neutralization of IL-10 (Fig 11E), suggesting an indirect effect that required synthesis of this cytokine. Lastly, we found that IFN-γ induced a STAT3-dependent IL-10 response in *in vitro* infected bone marrow-derived macrophages (Fig 11F) and *ex vivo* stimulated splenic macrophages from hamsters with VL (Fig 11G). Collectively, these data indicate that IFN-γ promotes disease in experimental VL in part through direct and indirect (via IL-10) activation of STAT3, which then acts on downstream genes (e.g. Arg1) that confer a permissive macrophage phenotype. The blockade of this process with STAT3 inhibitors, which are being extensively studies as non-cytotoxic chemotherapeutics for some cancers [112], identifies STAT3 as a candidate target for adjunctive host-directed therapy in VL.

IFN-γ is well-established as having a critical role in protection against *Leishmania* infection so our contrary finding of an infection-promoting effect deserves some contextual discussion. A number of previous studies in experimental models demonstrated that IFN-γ is required, but not sufficient, to protect against *Leishmania* infection [113]. The reason that the high endogenous IFN-γ production in the spleen is not protective in human VL has been an enigma. A clinical benefit of exogenous IFN-γ as adjunctive therapy was shown when it was combined with antileishmanial chemotherapy (pentavalent antimony) [114,115]. Since the anti-leishmanial activity of pentavalent antimony is mediated in part through modulating macrophage signaling [116], this drug may make the cells more responsive to IFN-γ-induced activation. Use of IFN-γ as monotherapy in VL was beneficial in some but not all patients [117], and the variable response was one of the reasons that IFN-γ was abandoned as a therapeutic agent for VL. *Ex vivo* cultures of splenic aspirates from patients with VL [118] showed that endogenous splenic IFN-γ was protective in most, but not all, subjects. In some subjects, the parasite burden decreased with IFN-γ neutralization, suggesting that It might play a pathological role. Collectively, these data suggest that a subset of patients fail to benefit from endogenous splenic IFN-γ expression and some are refractory to the effects of exogenous IFN-γ. Our data suggest a possible explanation for this enigma: that in some patients with VL, IFN-γ is part of a broad splenic proinflammatory response that drives an exuberant STAT3-dependent counter-regulatory response that promotes disease. Further investigation is needed to determine if this is the case.

## Conclusions

Genome-wide expression analysis revealed evidence of a broad inflammatory signature that included an extensive array of upregulated interferon response genes in the spleen during progressive VL. This type of gene expression would be expected to drive macrophages toward a M1 phenotype and protect against *Leishmania* [119]. However, M1 polarization was not dominant and IFN-γ paradoxically enhanced parasite growth in splenic macrophages. Importantly, the parasite-promoting effect of IFN-γ was more pronounced in splenic macrophages isolated later in the course of infection. This suggests that as VL progresses, splenic macrophages in VL are conditioned by the chronic inflammatory environment to respond to macrophage activation signals in an aberrant, pathological way that contributes to the progressive infection. Several mechanisms could account for this. First, the finding of fewer upregulated IFN-response genes in splenic macrophages relative to the whole spleen, including transcripts known to be induced in macrophages, suggests relative macrophage unresponsiveness to IFN-γ. The absence of NOS2 upregulation is likely a central determinant of ineffective parasite killing. Impaired IFN-γ signaling, which has been well-described in *in vitro* infected macrophages [120–124], is likely to contribute to impaired NOS2 expression during VL. Second, the co-expression of M1- and M2-associated transcripts, and the finding that IFN-γ induces Arg1, are suggestive of a misdirected signaling in splenic macrophages during VL. There is a growing body of evidence that Arg1 has a significant pathological role in *Leishmania* infection, including human VL [19,20,80–82,125,126]. Our data indicate that expression of Arg1 is not part of a conventional Th2-driven M2 macrophage phenotype, but identify a previously unrecognized mechanism of IFN-induced Arg1. This has significant bearing on the pathogenesis of VL so the mechanisms of IFN regulation of Arg1 in VL need further investigation. Third, parasite-derived signals and anti-inflammatory/regulatory cues (e.g. IL-10 and IL-21) [19,20] may impair macrophage effector function and lead to disease-promoting gene expression. Fourth, the massive interferon response in the spleen appears to have a counter-protective effect through initiation of an exuberant counter-regulatory response mediated via STAT3 and IL-10. The STAT3-dependent IFN-γ-induced Arg1 expression may paradoxically lead to impaired macrophage or T cell responses, as we and others have described previously in VL [19,20,80–82,125,126]. Lastly, the high expression of a broad array of chemokines in the spleen is likely to lead to accumulation of immature myeloid cell populations, which have some features consistent with myeloid-derived suppressor cells, that are less responsive to classical activation signals. Collectively, these data identify a number of molecules, pathways and transcription factors that contribute to the pathogenesis of VL. The STAT3 pathway in particular is an attractive target for adjunctive host-directed therapy for VL.

## Materials and Methods

### Ethics statement

The animals used in this study were handled in strict accordance with the recommendations in the Guide for the Care and Use of Laboratory Animals of the National Institutes of Health. The protocol was approved by the Institutional Animal Care and Use Committee of the University of Texas Medical Branch, Galveston, Texas (protocol number 1101004).

### Animal model and *Leishmania donovani* infection

6–8 week old outbred Syrian hamsters females (*Mesocricetus auratus*) were obtained from Harlan Laboratories. Hamsters were either uninfected or infected (n = 4–8 per group) with 1x10^6^ peanut agglutinin purified *Leishmania donovani* (MHOM/SD/001S-2D) metacyclic promastigotes by intracardial injection as we have described previously [127].

### Isolation of total spleen cells and splenic macrophages

Hamsters were sacrificed at 28 days post-infection by CO_2_ inhalation and the spleens were collected in complete DMEM (Gibco), supplemented with 2% fetal bovine serum (FBS), 1 mM Sodium pyruvate (Gibco), 1X MEM amino acids solution (Sigma), 0.02% v/v/ EDTA, 10 mM HEPES buffer (Cellgro) and 100 IU/mL Penicillin/100mg/mL Streptomycin solution (Cellgro). Adherent spleen cells were isolated after infiltrating the whole organ with the injection of 2 mL of Collagenase D (Sigma) at 2 mg/mL. The spleen tissue was cut into small pieces and incubated for 20 minutes at 37°C resuspended in the enzyme solution. The cell suspension and remaining tissue fragments were suspended in culture medium and gently passed using a syringe plug through a 100 μm cell strainer (B-D) to obtain a single cell suspension. Cells were cultured in a 75cm flasks and were allowed to adhere for 4 hr at 37°C and the non-adherent cells were removed after washing the monolayers with pre- warmed PBS (10 times), before the RNA was isolated.

### RNA isolation and RNA-seq library preparation and sequencing

For isolation of RNA from the whole spleen, it was cut in small pieces, resuspended in 2 mL of lysis buffer and homogenized to isolate RNA using the RNAqueus kit (Ambion-Life Technologies, CA) following the manufacturers protocol. Adherent splenic macrophages were similarly lysed and the RNA isolated. Libraries for deep sequencing were constructed from poly-A RNA isolated from the spleens or splenic macrophages of uninfected and 28-day *L*. *donovani*-infected hamsters (n = 4 per group) using the Illumina TruSeq RNA Sample Preparation kit. The library quality was confirmed by Agilent Bioanalyzer. Paired-end 50-base sequencing was performed using TruSeq SBS kit v3 (Illumina) on an Illumina HiSeq 1000.

### De novo transcriptome assembly

The quality of raw sequencing reads was checked using FastQC (v0.10.1) [128]. To avoid the contamination of pathogen sequences, we filtered out reads (using the FASTX Toolkit v0.0.13) that aligned to the *Leishmania donovani* BPK282A1 genome (NCBI BioProject PRJEA61817) [29] using Bowtie 2 (v2.0.0-beta5) [129] under default options. To reduce the effect of low quality reads, we further filtered out artifacts and reads having a phred score <28 in more than 10% of nucleotides using FASTX Toolkit (v0.0.13). Both forward and reverse reads were removed if any of them failed to pass the filters. To obtain a complete transcriptome, we used two steps. First, the cleaned sequencing reads from different spleen samples were pooled together and *de novo* assembled using Trinity software [23]. Second, the resulting transcriptome was combined with all cleaned reads from hamster spleen and splenic macrophages and the CHO-K1 RefSeq genome [28] to perform a second *de novo* assembly using BRANCH [22]. Both assembling steps were run in collaboration with Texas Advanced Computing Center (TACC) at the University of Texas at Austin.

### BLAST filtering

We first created a customized reference library using *Rattus norvegicus* (Rnor_5.0.73) and *Mus musculus* (GRCm38.73) genomes. We then used BLAST (v 2.2.28+) [30] to align each transcript generated from BRANCH against the customized library to assign it a gene name based upon sequence similarity. An E-value cutoff of <1e-3 was used. Additionally, we compared the Trinity transcripts with the CHO-K1 Ref Seq genome.

### RNA-Seq differential analysis

The spleen tissue and splenic macrophage RNA samples were collected and sequenced in two different experiments, so we analyzed them separately to avoid the batch effects. All the non-*Leishmania*-like raw sequencing reads were first mapped to our *de novo* reference genome using Bowtie2 (v2.1.0) with default options, but allowing one read to map to as many as 500 different transcripts. We then measured the expression abundance, count of reads mapped to each transcript, using the software eXpress. The effective counts were recommended for RNA-Seq differential expression analysis because they correct biases caused by multiple hits and mismatches in alignment. To identify differentially expressed transcripts in each experiment, 3 different approaches were applied: exact tests of Robinson and Smyth [130] and generalized linear models with the likelihood ratio test using the BioConductor R package EdgeR [35], and the Wald test using DESeq2 [36]. Only transcripts with at least 1 count per million in at least 3 out of 4 samples in control or experimental group were included in the analysis. A transcript was considered differentially expressed only when it was identified by all three different approaches. The transcriptome data have been deposited in NCBI's Gene Expression Omnibus [54] and are accessible through GEO Series accession number GSE91187 (http://www.ncbi.nlm.nih.gov/geo/query/acc.cgi?acc=GSE91187).

#### Ingenuity Pathway Analysis (IPA)

We evaluated the functional significance of differentially expressed transcripts in hamsters with VL using Ingenuity Pathway analysis (IPA) software (http://www.ingenuity.com) and gene set enrichment analysis [131]. We uploaded data sets (those that met false discovery rate (FDR) cut-offs of either FDR < 0.001 or FDR < 0.01) into IPA, and mapped them to the pathways available in the program to identify the most significant canonical pathways.

#### Gene Set Enrichment Analysis (GSEA)

We performed GSEA via Broad Institute (http://www.broadinstitute.org/gsea) using MSigDB C5: GO gene set collection (1379 gene set available) (v4.0). The GSEA determines the significance of a pre-defined gene set by comparing the correlation between their expression and the class distinction to other random situations [132]. We carried out 1000 random gene set permutations because each group has 4 samples, and the significance threshold was set at FDR<0.1.

### Validation of gene expression by quantitative RT-PCR

RNA samples were DNase treated with TURBO DNA-free kit (Ambion) and quantified using a NanoDrop Spectrophotometer (Thermo Scientific) and maintained at -80°C until used. 250–500 ng of RNA were used for cDNA synthesis using the high capacity cDNA reverse transcription kit (Applied Biosystems). Gene expression was determined by SYBR green (Applied Biosystems) PCR using primers whose sequences were reported previously [20,21] or are shown in S6 Table, at a final concentration of 300–500 nM. With the exception of CCL17, SOCS1, and IRG1 primers were designed to span an intron, and were confirmed by analysis of dissociation curves to not generate primer dimers. Data was analyzed using the comparative Ct method, relative to uninfected control spleen or macrophages, and with the 18S rRNA gene as the normalizer.

### Gene expression by *in situ* amplification and fluorescence hybridization

The expression of M1- and M2-associated transcripts in splenic macrophages was determined at the single cell level using the QuantiGene ViewRNA ISH cell Assay (Affymetrix, Santa Clara, CA). RNA was visualized following the manufacturer protocol with the exception that MOWIOL (Sigma, ST Louis, MO) was used as mounting media. Spleen cells from infected hamsters and uninfected controls were allowed to attach to poly-L-lysine coated glass cover slips for 3 hr. After-fixation with 4% formaldehyde, adherent cells on coverslips were incubated with 1× detergent solution and digested with a protease solution (1:4000). Cells were subsequently hybridized with specific probes sets (1:100 in diluent QF), conjugated to a specific fluorescent dye with different excitation wavelengths. Probes were designed to target the hamster IDO1 (type1 fluorophore-550nm), CXCL9 (type 1 fluorophore-550nm), Arg1 (type 6 fluorophore-650nm) and CD68 mRNA (type 4 fluorophore-488nm) using the accession numbers (XM_005066560, MH01025X1B04, NM_001281645 and XM_005067542), respectively (Affymetrix). Cells were then hybridized with pre-amplifier (1:25 in amplifier diluent QF) and amplifier (1:25 in amplifier diluent QF) mix solutions. All hybridization steps were carried out at 40°C and were followed by three washes with washing buffer. All hybridized slides were examined by confocal microscopy and the number of fluorescent cells counted manually. Nuclei were stained with a 1X working DAPI solution in PBS. Cells incubated with no probe were used as negative controls.

### *In vitro* and *ex vivo* macrophage cultures

To determine effect of IFNs in macrophages, splenic macrophages were obtained as described above and BMDM were generated as we described previously [20]. Cells were infected with stationary phase L. donovani promastigotes (5 parasites per macrophage) as described previously [20]. Uninfected or *L*. *donovani* infected macrophages were stimulated with recombinant hamster IFN-γ expressed in CHO cells (10% v/v supernatant) or CHO cells transfected with empty vector (mock supernatant control) as described [16], or with recombinant IFN-α (Universal Type I Interferon, PBL Interferon Source) at 100 U/mL. Infections and treatments were carried out for 24 hrs. For treatment with the STAT3 inhibitor, hamster BMDM or splenic macrophages were treated with 25–100 μM STAT3 inhibitor (S31-201, Cayman) or DMSO vehicle control for 30 min before stimulation with recombinant hamster IFN-γ. The viability of cells after treatment was >95% (CellTiter Glo, Promega).

### Determination of parasite burden

The parasite burden in infected macrophages was determined by quantitation of *Leishmania* 18S **r**RNA expression by qPCR. RNA was extracted and reverse-transcribed [20] and the *Leishmania* 18S rRNA amplified and quantified by SYBR-Green reaction (Bio-Rad). The primers used were: For: 5’- CCAAAGTGTGGAGATCGAAG-5’ and Rev: 5’- GGCCGGTAAAGGCCGAATAG-3’. The number of parasites was determined by extrapolation from a standard curve generated from purified splenic amastigotes, or by the relative expression determined by comparison to mock-infected cells (background Ct value).

### Immunoblotting

Phosphorylation of STAT-1 and STAT-3 was determined by immunoblotting of splenic macrophages obtained at different times after infection of hamsters with *L*. *donovani* as described [20]. The following antibodies were used: p-STAT1 (9171, Cell Signaling), p-STAT3 (9145, Cell Signaling), GAPDH (Clone 6C5, Millipore) and STAT3 (Sc-80910, SantaCruz). Bands were captured with a Chemi X T4 camera (G BOX, SynGene) and relative band intensity calculated by densitometry analysis with the softGeneTools Analysis Software (Syngene).

### STAT3 reporter assay

Hamster BHK-21 cell line was transiently transduced with lentiviral particles carrying a STAT-3 luciferase reporter construct (Cignal Lenti Reporter, Qiagen). Cells (5,000 cells/100 μL) were transduced at 20 MOI in 8 μg/mL polybrene in DMEM with 10% FBS. At 16 hrs post-transduction the medium was changed, and after 72h cells were exposed or not to *L*. *donovani* promastigotes (1:5), treated with or without recombinant hamster IFN-γ, and pre-treated with or without 2 μg/mL of anti-IL-10 antibody (mouse/rat anti-IL-10, R&D, AF519, R&D). Activity of the STAT-3 reporter was expressed as relative luciferase activity per number of cells determined with cell titer Glo (Promega).

### Determination of plasma endotoxin concentration

Bacterial endotoxin level was determined by the Limulus Amebocyte Lysate (LAL) method (Pierce LAL Chromogenic Endotoxin Quantitation Kit, Thermo Scientific) in serum samples from uninfected or infected hamsters according the manufacturer instructions.

### Statistical analysis

We implemented “trimmed mean for M-values” (TMM) method for normalization and calculated the significance using tagwise dispersion in the RNA differential analysis. The Benjamin–Hochberg procedure was applied to gain the False Discovery Rate (FDR) from p values for multiple tests. Differences in mRNA expression determined by qRT-PCR between non-infected and 28 day infected animals were analyzed by two tail Mann-Whitney test or two tail unpaired t-test using GraphPad Prism version 5.01 for windows, GraphPad Software, San Diego California USA (www.graphpad.com). In instances where groups were compared, ANOVA with a post hoc correction for multiple comparisons (Bonferroni or Tukey) was applied. All other statistical analyses are as described in the body of the paper or figure legends.

## Supporting Information

S1 Fig Quality assessment of RNA sequencing data. **(A)** Quality (Phred) scores at each base position. **(B)** Quality (Phred) score distribution across all sequences. **(C)** GC distribution over all sequences compared to theoretical distribution. **(D)** Percentage of unidentified nucleotides (N content) across all bases.
(PDF)

S2 Fig Characterization of RNA sequences and differentially expressed transcripts. Clustering of samples within groups shown by Principal Component Analysis of RNA sequences from uninfected and infected spleen tissue **(A)** and splenic macrophages **(B). (C)** Venn diagram of the number of differentially expressed transcripts in spleen and splenic macrophages (MΦ) from hamsters with VL determined by exact test (FET), generalized linear model with likelihood ratio test (LRT), and Wald test using a false discovery rate (FDR) of <0.001 as the cutoff. Only transcripts with at least 1 count per million in at least 3 out of 4 samples in control or experimental group were included in the analysis. A transcript was considered differentially expressed only when it was identified by all three different approaches.
(PDF)

S3 Fig Canonical pathway analysis and Gene Set Enrichment Analysis (GSEA) of differentially expressed transcripts. **(A)** Top 50 canonical pathways (CPs) in the spleen (blue bars) compared to splenic macrophages (gray bars). **(B)** Top 50 canonical pathways (CPs) in splenic macrophages (gray bars) compared to spleen tissue (blue bars). **(C)** Top 10 gene sets identified in spleen and splenic macrophages determined by Gene Set Enrichment Analysis (GSEA) and Gene Ontology (GO) analysis. The N Enrichment Score (NES), nominal p value, and False Discovery Rate (FDR) are shown for each gene set in the table. A pictorial representation of the inflammatory response, cytokines and chemokines, and collagen and extracellular matrix gene sets is shown.
(PDF)

S4 Fig Serum endotoxin levels in uninfected and *L*. *donovani* infected hamsters. Blood was collected from euthanized uninfected (n = 21) or 28-day infected (n = 10) hamsters by terminal cardiac puncture. After clotting, the serum was separated and endotoxin concentration determined by ELISA. Data are expressed as a single endotoxin unit (EU) value per animal with the median and 25^th^ and 75^th^ percentiles shown as a horizontal lines.
(PDF)

S5 Fig Network analysis of differentially expressed transcripts of type I (IFNα/β) and type II (IFN-γ) interferon and interferon-response genes in the spleen tissue of hamsters with VL. Transcripts identified as significantly upregulated (FDR<0.01) were loaded into IPA and compared against a manually curated set of IFN-responsive genes. The network connections of those transcripts known to interact with either interferon alpha, beta, or gamma (IFN-α, IFN-β, or IFN-γ) is shown.
(PDF)

S1 Table Cell markers used to evaluate lineage of adherent spleen cells. (PDF)

S2 Table Expression of cytokines, chemokines and Innate Immune Receptors in infected spleen tissue and splenic macrophages. (PDF)

S3 Table TFs predicted to be activated/inhibited in infected spleen tissue and splenic macrophages. (PDF)

S4 Table Expression of M1- and M2-associated genes in infected spleen tissue and splenic macrophages. (PDF)

S5 Table Expression of Hamster Interferon Response Genes in infected spleen tissue and splenic macrophages. (PDF)

S6 Table Primer sequences used for real time RT-PCR assays. (PDF)
