# Supplementary material for: Transcriptional Profiling in Experimental Visceral Leishmaniasis Reveals a Broad Splenic Inflammatory Environment that Conditions Macrophages toward a Disease-Promoting Phenotype
Source: PLoS Pathog. 2017 Jan 31;13(1):e1006165. doi: 10.1371/journal.ppat.1006165 (PMC5283737; doi:10.1371/journal.ppat.1006165)
Supplement: S1 Fig — (A) Quality (Phred) scores at each base position. (B) Quality (Phred) score distribution across all sequences. (C) GC distribution over all sequences compared to theoretical distribution. (D) Percentage of unidentified nucleotides (N content) across all bases. (PDF) [file ppat.1006165.s001.pdf]

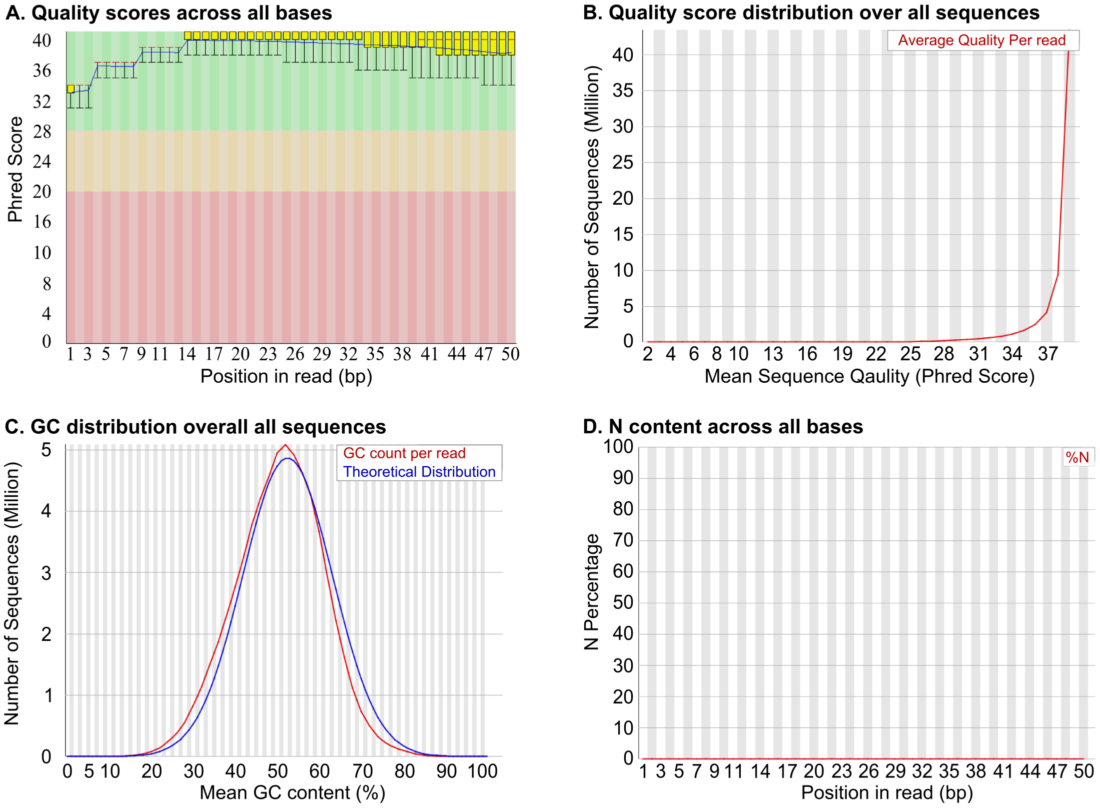
A. Quality scores across all bases
Phred Score
40
36
32
28
24
20
16
10
8
4
0
1 3 5 7 9 11 14 17 20 23 26 29 32 35 38 41 44 47 50
Position in read (bp)
B. Quality score distribution over all sequences
Average Quality Per read
Number of Sequences (Million)
40
35
30
25
20
15
10
5
0
2 4 6 8 10 13 16 19 22 25 28 31 34 37
Mean Sequence Qaulity (Phred Score)
C. GC distribution overall all sequences
GC count per read
Theoretical Distribution
Number of Sequences (Million)
5
4
3
2
1
0
0 5 10 20 30 40 50 60 70 80 90 100
Mean GC content (%)
D. N content across all bases
%N
N Percentage
100
90
80
70
60
50
40
30
20
10
0
1 3 5 7 9 11 14 17 20 23 26 29 32 35 38 41 44 47 50
Position in read (bp)
