# Supplementary figures and images for: Transcriptional Profiling in Experimental Visceral Leishmaniasis Reveals a Broad Splenic Inflammatory Environment that Conditions Macrophages toward a Disease-Promoting Phenotype
Source: PLoS Pathog. 2017 Jan 31;13(1):e1006165. doi: 10.1371/journal.ppat.1006165 (PMC5283737; doi:10.1371/journal.ppat.1006165)

**A. Spleen tissue MDS plot**

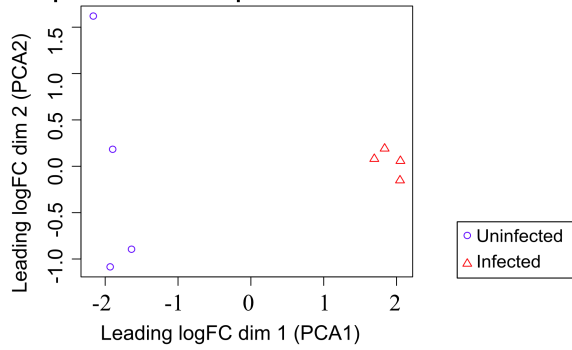

**B. Splenic MΦ MDS plot**

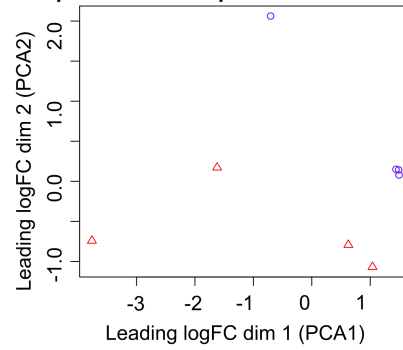

**C. RNA-Seq analysis results**

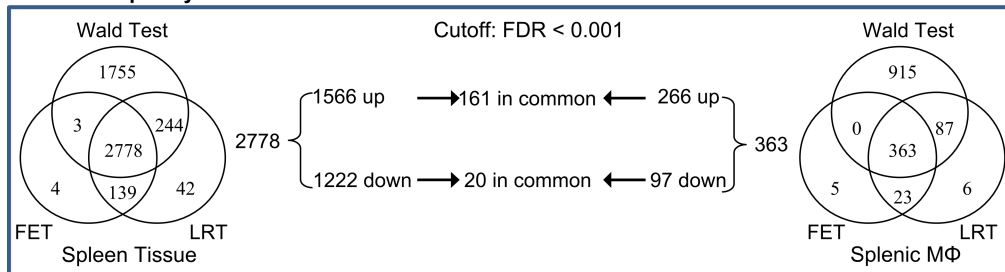

Supplement: S2 Fig — Clustering of samples within groups shown by Principal Component Analysis of RNA sequences from uninfected and infected spleen tissue (A) and splenic macrophages (B). (C) Venn diagram of the number of differentially expressed transcripts in spleen and splenic macrophages (MΦ) from hamsters with VL determined by exact test (FET), generalized linear model with likelihood ratio test (LRT), and Wald test using a false discovery rate (FDR) of <0.001 as the cutoff. Only transcripts with at least 1 count per million in at least 3 out of 4 samples in control or experimental group were included in the analysis. A transcript was considered differentially expressed only when it was identified by all three different approaches. (PDF) [file ppat.1006165.s002.pdf]

### A. Top 50 CP in spleen tissue

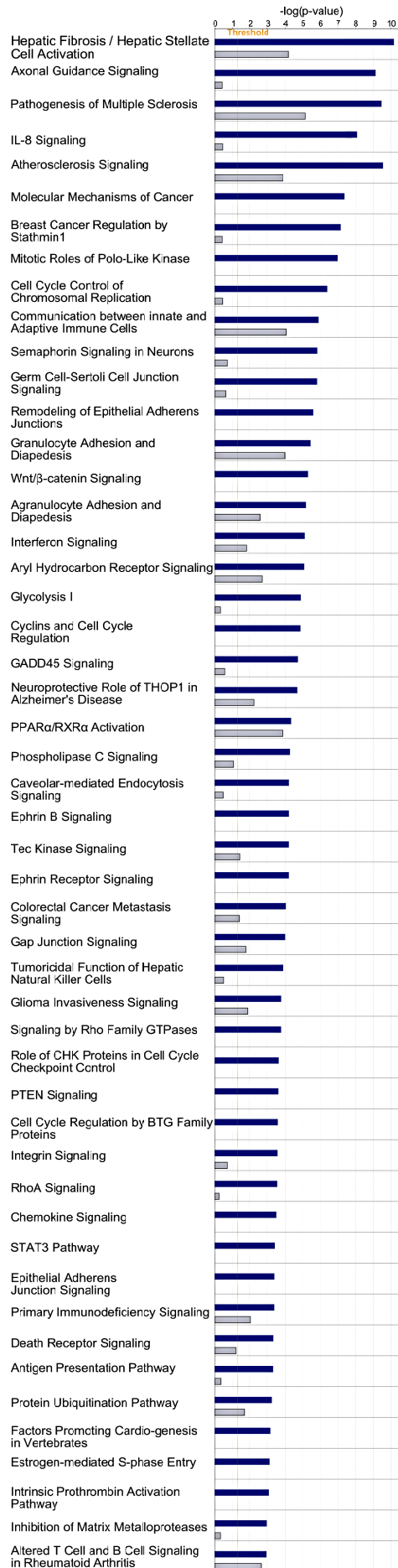

### B. Top 50 CP in splenic MΦ

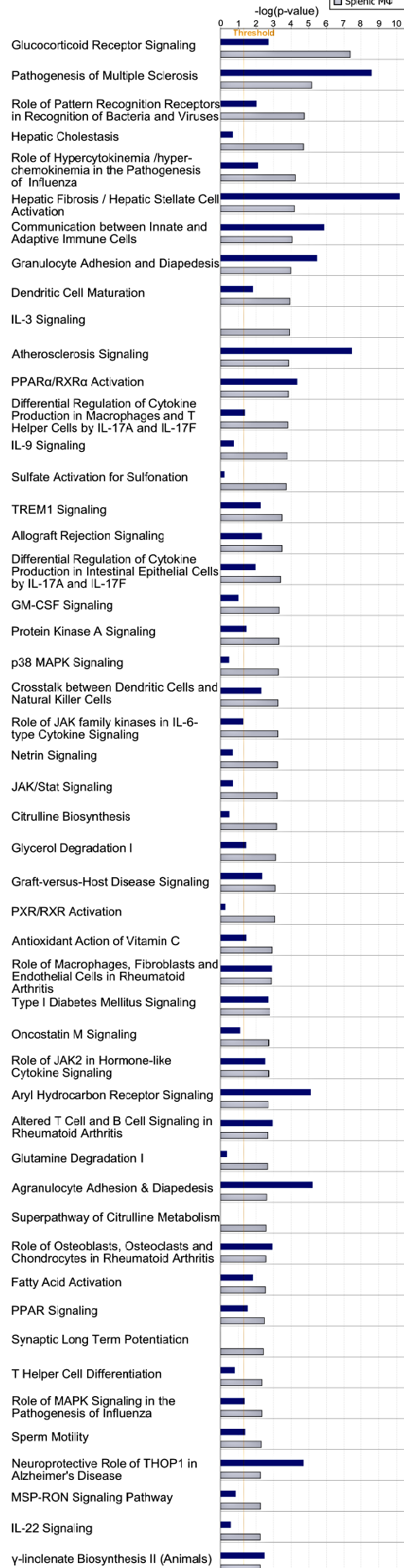

C. GSEA results

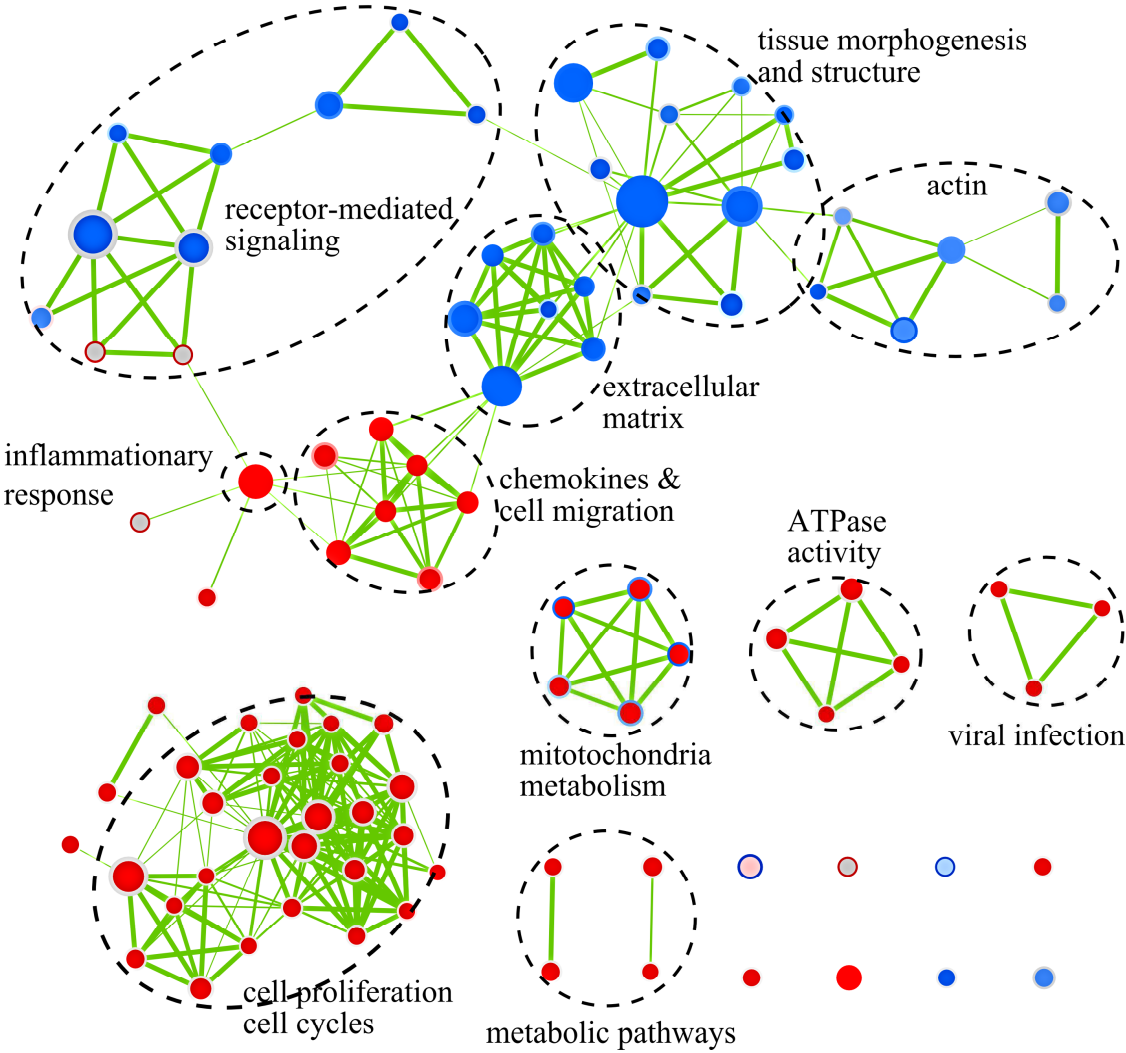

Supplement: S3 Fig — (A) Top 50 canonical pathways (CPs) in the spleen (blue bars) compared to splenic macrophages (gray bars). (B) Top 50 canonical pathways (CPs) in splenic macrophages (gray bars) compared to spleen tissue (blue bars). (C) Top 10 gene sets identified in spleen and splenic macrophages determined by Gene Set Enrichment Analysis (GSEA) and Gene Ontology (GO) analysis. The N Enrichment Score (NES), nominal p value, and False Discovery Rate (FDR) are shown for each gene set in the table. A pictorial representation of the inflammatory response, cytokines and chemokines, and collagen and extracellular matrix gene sets is shown. (PDF) [file ppat.1006165.s003.pdf]

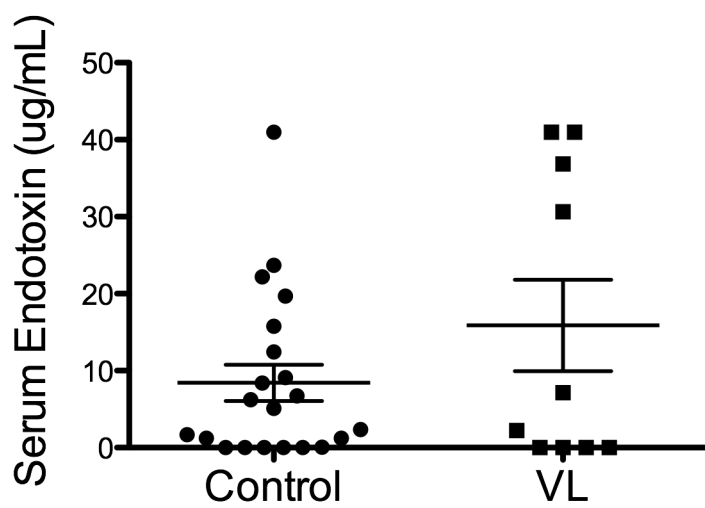

Supplement: S4 Fig — Blood was collected from euthanized uninfected (n = 21) or 28-day infected (n = 10) hamsters by terminal cardiac puncture. After clotting, the serum was separated and endotoxin concentration determined by ELISA. Data are expressed as a single endotoxin unit (EU) value per animal with the median and 25th and 75th percentiles shown as a horizontal lines. (PDF) [file ppat.1006165.s004.pdf]

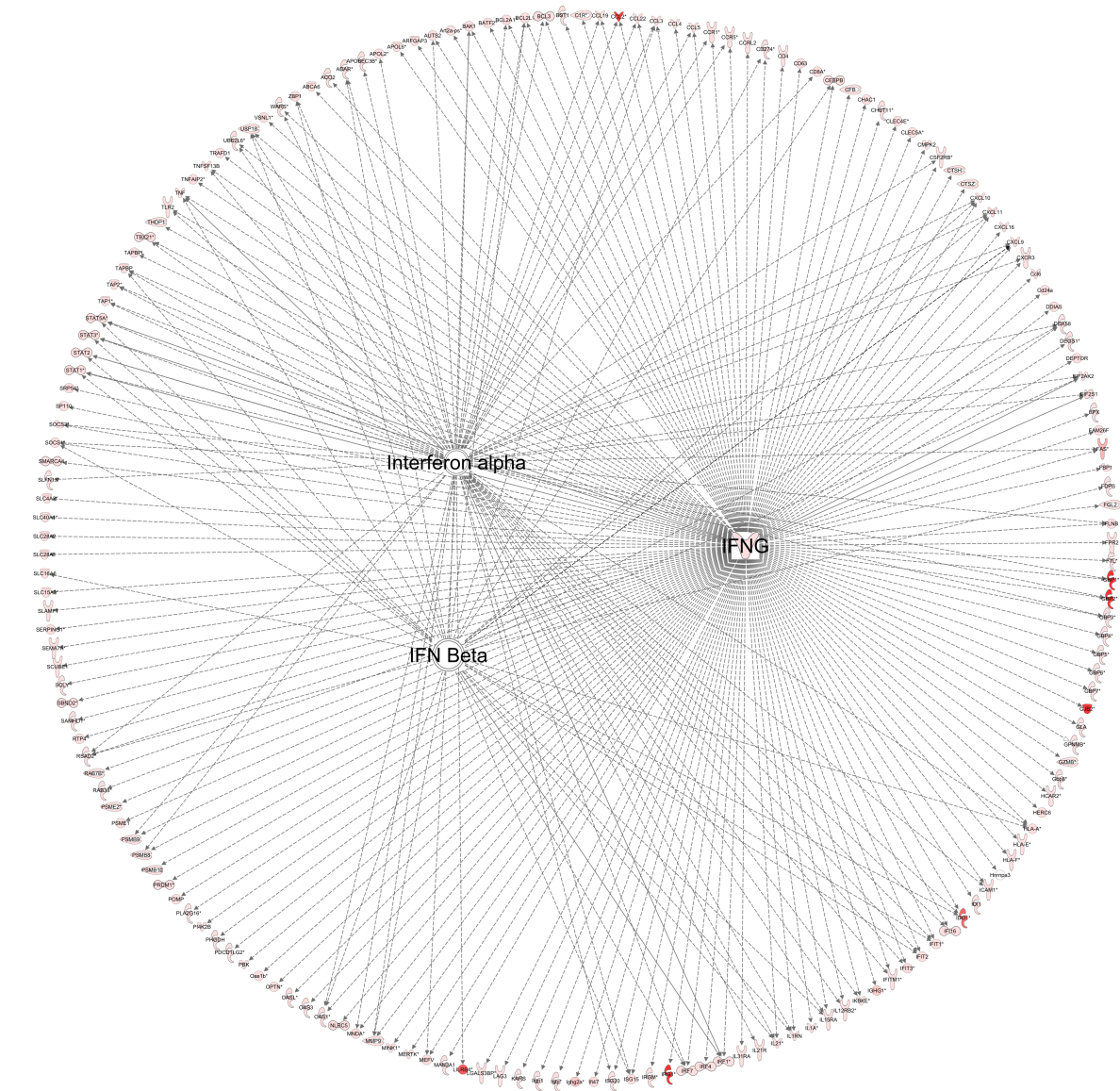

Supplement: S5 Fig — Transcripts identified as significantly upregulated (FDR<0.01) were loaded into IPA and compared against a manually curated set of IFN-responsive genes. The network connections of those transcripts known to interact with either interferon alpha, beta, or gamma (IFN-α, IFN-β, or IFN-γ) is shown. (PDF) [file ppat.1006165.s005.pdf]
