# Supplementary material for: Transcriptional Profiling in Experimental Visceral Leishmaniasis Reveals a Broad Splenic Inflammatory Environment that Conditions Macrophages toward a Disease-Promoting Phenotype
Source: PLoS Pathog. 2017 Jan 31;13(1):e1006165. doi: 10.1371/journal.ppat.1006165 (PMC5283737; doi:10.1371/journal.ppat.1006165)
Supplement: S1 Table — (PDF) [file ppat.1006165.s006.pdf]

Table S1. Cell markers used to evaluate lineage of adherent spleen cells [1-8]

Cell lineage	Gene symbol	Entrez Gene Name	Blast results		Mean CPM		Ratio	*p* value
			Score	EValue	Spleen	Spleen MΦ		
Monocyte / Macrophage	Emr1	Egf-like module containing, mucin-like, hormone receptor-like 1	2847	0	325.39	471.1	1.45	<0.001
	Cd163	CD163 molecule	2477	0	3.12	4.57	1.46	0.338
	Slc11a1	Solute carrier family 11 (proton-coupled divalent metal ion transporter), member 1	2185	0	60.45	103.59	1.71	0.008
	Mertk	c-mer proto-oncogene tyrosine kinase	4695	0	78.41	150.7	1.92	0.001
	Cd14	CD14 molecule	1213	0	72.2	141.1	1.95	0.010
	Mafb	v-maf avian musculoaponeurotic fibrosarcoma oncogene homolog B	3416	0	256.66	529.34	2.06	0.002
	Fcgr1	Fc receptor, IgG, high affinity I	1577	0	60.44	148.34	2.45	0.003
	Cd68	CD68 molecule	1261	0	152.16	795.04	5.23	<0.001
	Spi1	Spleen focus forming virus (SFFV) proviral integration oncogene	2044	0	94.57	554.06	5.86	<0.001
	Csf1r	Colony stimulating factor 1 receptor	183	3E-45	0.2	1.51	7.69	0.104
	Lgals3	Lectin, galactoside-binding, soluble, 3	884	0	228.71	1881.62	8.23	0.018
	Mmp12	matrix metallopeptidase 12 (macrophage elastase)	1535	0	859.49	19724.78	22.95	<0.001
	Mpeg1	macrophage expressed 1	3438	0	302.21	136.44	-2.21	0.011
Dendritic cell	Kit	v-kit Hardy-Zuckerman 4 feline sarcoma viral oncogene homolog	4776	0	16.7	6.83	-2.45	0.010
	Zbtb46	zinc finger and BTB domain containing 46	6574	0	31.65	7.72	-4.1	0.008
	Flt3	fms-related tyrosine kinase 3	4691	0	15.77	2.57	-6.14	<0.001
	Dpp4	dipeptidyl-peptidase 4	5707	0	28.46	2.25	-12.65	0.007
	Btla	B and T lymphocyte associated	1510	0	87.34	1.25	-70.02	<0.001
Neutrophil	Neu2	sialidase 2 (cytosolic sialidase)	1860	0	2.55	0.81	-3.15	0.123
	Ltf	lactotransferrin	2569	0	9.5	0.38	-25.21	0.005
	Prtn3	proteinase 3	1012	0	0.81	0.01	-116.13	0.208
	Mpo	myeloperoxidase	2980	0	2.69	0	-Inf	0.021
Fibroblast	Acta1	actin, alpha 1, skeletal muscle	1339	0	1.08	1.07	-1.01	0.975
	Vim	vimentin	2940	0	813.45	256.73	-3.17	<0.001
	S100a4	S100 calcium binding protein A4	628	5E-179	11.69	1.42	-8.24	0.001
	P4hb	prolyl 4-hydroxylase, beta polypeptide	3231	0	256.51	343.85	1.34	0.012
Lymphocyte	Cd247	CD247 molecule	1178	0	25.83	6.44	-4.01	<0.001
	Cd69	CD69 molecule	1314	0	22.17	5.48	-4.04	0.005
	Cd8a	CD8a molecule	581	1E-164	8.58	1.78	-4.81	0.007
	Tbx21	T-box 21	2953	0	11.72	0.66	-17.67	<0.001
	Gata3	GATA binding protein 3	3261	0	4.56	0.2	-22.57	<0.001
	Cd19	CD19 molecule	2156	0	31.78	1.25	-25.4	0.002
	Cd3d	CD3d molecule, delta (CD3-TCR complex)	930	0	50.49	1.46	-34.57	0.003
	Cd28	CD28 molecule	1631	0	10.48	0.3	-35.17	<0.001
	Cd3e	CD3e molecule, epsilon (CD3-TCR complex)	955	0	53.16	1.35	-39.36	<0.001
	Cd22	CD22 molecule	2253	0	68.44	1.48	-46.14	<0.001
	Cd27	CD27 molecule	1021	0	21.24	0.42	-50.92	<0.001
	Cd4	CD4 molecule	1344	0	25.24	0.39	-64.91	0.001
	Cd8b	CD8b molecule	969	0	9.5	0.13	-75.37	0.005

References

1. Shapiro SD, Kobayashi DK, Ley TJ (1993) Cloning and characterization of a unique elastolytic metalloproteinase produced by human alveolar macrophages. J Biol Chem 268: 23824-23829.
2. Beyer M, Mallmann MR, Xue J, Staratschek-Jox A, Vorholt D, et al. (2012) High-Resolution Transcriptome of Human Macrophages. PLoS ONE 7: e45466.
3. Lau SK, Chu PG, Weiss LM (2004) CD163: a specific marker of macrophages in paraffin-embedded tissue samples. Am J Clin Pathol 122: 794-801.

4. Vidal SM, Malo D, Vogan K, Skamene E, Gros P (1993) Natural resistance to infection with intracellular parasites: isolation of a candidate for Bcg. Cell 73: 469-485.
5. Murray PJ, Wynn TA (2011) Protective and pathogenic functions of macrophage subsets. Nat Rev Immunol 11: 723-737.
6. Franklin RA, Liao W, Sarkar A, Kim MV, Bivona MR, et al. (2014) The cellular and molecular origin of tumor-associated macrophages. Science 344: 921-925.
7. Jersmann HP (2005) Time to abandon dogma: CD14 is expressed by non-myeloid lineage cells. Immunol Cell Biol 83: 462-467.
8. Perussia B, Starr S, Abraham S, Fanning V, Trinchieri G (1983) Human natural killer cells analyzed by B73.1, a monoclonal antibody blocking Fc receptor functions. I. Characterization of the lymphocyte subset reactive with B73.1. Journal of immunology (Baltimore, Md : 1950) 130: 2133-2141.
