# Supplementary material for: Transcriptional Profiling in Experimental Visceral Leishmaniasis Reveals a Broad Splenic Inflammatory Environment that Conditions Macrophages toward a Disease-Promoting Phenotype
Source: PLoS Pathog. 2017 Jan 31;13(1):e1006165. doi: 10.1371/journal.ppat.1006165 (PMC5283737; doi:10.1371/journal.ppat.1006165)
Supplement: S2 Table — (PDF) [file ppat.1006165.s007.pdf]

Table S2. Cytokines, chemokines and Innate Immune Receptors

Symbol	Entrez Gene Name	Blast Result		Spleen Infection		Splenic MΦ Infection	
		Score	Evalue	FC	FDR	FC	FDR
CCL11	chemokine (C-C motif) ligand 11	823	0	20.48	<0.001	--	--
CCL17	chemokine (C-C motif) ligand 17	383	4E-105	70.91	<0.001	4.53	0.009
CCL19	chemokine (C-C motif) ligand 19	379	2E-104	1.74	0.033	--	--
CCL2	chemokine (C-C motif) ligand 2	643	0	25.17	<0.001	1.36	0.577
CCL21	chemokine (C-C motif) ligand 21	881	0	-2.01	0.001	1.13	0.909
CCL22	chemokine (C-C motif) ligand 22	1023	0	4.01	0.001	1.26	0.743
CCL3	chemokine (C-C motif) ligand 3	623	4E-177	15.46	<0.001	3.28	<0.001
CCL4	chemokine (C-C motif) ligand 4	632	7E-180	11.72	<0.001	1.32	0.245
CCL5	chemokine (C-C motif) ligand 5	607	3E-172	16.3	<0.001	1.45	0.495
CCL6	chemokine (C-C motif) ligand 6	760	0	1.57	<0.001	2.39	0.002
CCL7	chemokine (C-C motif) ligand 7	731	0	32.4	<0.001	3.14	0.02
CCL8	chemokine (C-C motif) ligand 8	360	5E-98	120.48	<0.001	17.22	<0.001
CCL9	chemokine (C-C motif) ligand 9	747	0	-2.07	0.019	-1.42	0.727
CCR1	chemokine (C-C motif) receptor 1	1894	0	4.2	<0.001	2.09	<0.001
CCR1L1	chemokine (C-C motif) receptor 1-like 1	861	0	4.2	<0.001	2.09	<0.001
CCR2	chemokine (C-C motif) receptor 2	1296	0	1.14	0.638	2.7	0.063
CCR3	chemokine (C-C motif) receptor 3	2892	0	-4.05	<0.001	1.49	0.04
CCR5	chemokine (C-C motif) receptor 5 (gene/pseudogene)	1737	0	19.83	<0.001	2.38	<0.001
CCR7	chemokine (C-C motif) receptor 7	1743	0	-2.43	<0.001	-1.15	0.703
CCRL2	chemokine (C-C motif) receptor-like 2	1802	0	2.5	<0.001	-1.24	0.53
CSF2RB	colony stimulating factor 2 receptor, beta, low-affinity (granulocyte-macrophage)	2803	0	2.57	<0.001	2.53	<0.001
CXCL10	chemokine (C-X-C motif) ligand 10	618	2E-175	12.86	<0.001	1.28	0.365
CXCL11	chemokine (C-X-C motif) ligand 11	455	1E-126	104.37	<0.001	5.13	<0.001
CXCL12	chemokine (C-X-C motif) ligand 12	2778	0	-2.72	<0.001	1.53	0.454
CXCL14	chemokine (C-X-C motif) ligand 14	1584	0	-6.91	<0.001	--	--
CXCL16	chemokine (C-X-C motif) ligand 16	964	0	1.8	<0.001	1.08	0.839
CXCL2	chemokine (C-X-C motif) ligand 2	693	0	678.8	<0.001	-1.46	0.725
CXCL3	chemokine (C-X-C motif) ligand 3	181	2E-44	34.01	0.001	3.46	0.146
CXCL5	chemokine (C-X-C motif) ligand 5	699	0	94.92	<0.001	2.71	0.188
CXCL9	chemokine (C-X-C motif) ligand 9	1067	0	29.35	<0.001	4.39	<0.001
CXCR3	chemokine (C-X-C motif) receptor 3	1954	0	3.59	<0.001	--	--
CXCR6	chemokine (C-X-C motif) receptor 6	1710	0	13.62	<0.001	--	--
CX3CR1	CX3C chemokine receptor 1	1615	0	-2.49	0.048	--	--
IFNG	interferon, gamma	706	0	52.15	<0.001	11.12	<0.001
IL10	interleukin 10	1397	0	-1.27	0.611	1.51	0.051
IL10RA	interleukin 10 receptor, alpha	2839	0	1.12	0.529	2.42	0.001
IL15RA	interleukin 15 receptor, alpha	1034	0	8.12	<0.001	3.08	<0.001
IL18	interleukin 18 (interferon-gamma-inducing factor)	664	0	-1.81	0.002	1.44	0.268
IL1A	interleukin 1, alpha	1642	0	5.37	<0.001	1.21	0.632
IL1B	interleukin 1, beta	1294	0	-2.09	<0.001	1.95	<0.001
IL1R1	interleukin 1 receptor, type I	2870	0	-1.75	<0.001	1.13	0.91
IL1RL1	interleukin 1 receptor-like 1	2176	0	-3.22	0.002	1.02	0.984
IL21	interleukin 21	1604	0	68.93	<0.001	--	--
IL21R	interleukin 21 receptor	2174	0	2.01	<0.001	2.33	0.002
IL24	interleukin 24	338	3E-91	-3.21	<0.001	--	--
IL6	interleukin 6 (interferon, beta 2)	1074	0	--	--	5.04	0.007
PPBP	pro-platelet basic protein (chemokine (C-X-C motif) ligand 7)	650	0	-2.02	<0.001	1.13	0.766
SFPI1	spleen focus forming virus (SFFV) proviral integration oncogene	2044	0	1.38	0.002	-1.42	0.239
TGFBR2	transforming growth factor, beta receptor II (70/80kDa)	4800	0	-1.49	<0.001	-1.13	0.719
TLR2	Toll-Like Receptor 2	2839	0	3.5	<0.001	-1.2	0.415
TLR3	Toll-Like Receptor 3	4006	0	-2.4	<0.001	-1.7	0.433
TLR4	Toll-Like Receptor 4	3427	0	1.6	<0.001	2.4	<0.001
TNF	tumor necrosis alpha	1076	0	6.8	<0.001	-3.03	<0.001
TNFAIP2	tumor necrosis factor, alpha-induced protein 2	2224	0	3.76	<0.001	-1.3	0.357
TNFAIP3	tumor necrosis factor, alpha-induced protein 3	4969	0	2.83	<0.001	1.46	0.047
