# Supplementary material for: Transcriptional Profiling in Experimental Visceral Leishmaniasis Reveals a Broad Splenic Inflammatory Environment that Conditions Macrophages toward a Disease-Promoting Phenotype
Source: PLoS Pathog. 2017 Jan 31;13(1):e1006165. doi: 10.1371/journal.ppat.1006165 (PMC5283737; doi:10.1371/journal.ppat.1006165)
Supplement: S3 Table — (PDF) [file ppat.1006165.s008.pdf]

Table S3.

A. TFs predicted to be activated/inhibited in infected spleen tissue

Symbol	Entrez Gene Name	Blast Results		Differential Expression		IPA Prediction	
		Score	EValue	FC	FDR	Status	p-value
AHR	aryl hydrocarbon receptor	3710	0	-1.29	0.079	Activated	<0.001
Ahr-aryl hydrocarbon	--	NA	NA	--	--	Activated	0.042
ARNT	aryl hydrocarbon receptor nuclear translocator	2832	0	1.31	0.693	Activated	<0.001
ATF4	activating transcription factor 4	1952	0	1.31	0.014	Activated	<0.001
ATF6	activating transcription factor 6	3360	0	2.14	<0.001	Activated	0.005
BCL10	B-cell CLL/lymphoma 10	1314	0	1.06	0.817	Activated	0.003
Cbp/p300	Cbp/p300 group	1333	0	-1.02	0.947	Activated	0.003
DDIT3	DNA-damage-inducible transcript 3	210	3E-53	--	--	Activated	0.048
E2f	E2F group	NA	NA	--	--	Activated	<0.001
E2F1	E2F transcription factor 1	1921	0	1.50	0.134	Activated	<0.001
ESRRA	estrogen-related receptor alpha	3328	0	-1.12	0.474	Activated	<0.001
ESRRG	estrogen-related receptor gamma	NA	NA	--	--	Activated	<0.001
HHEX	hematopoietically expressed homeobox	1768	0	-1.54	<0.001	Activated	<0.001
HIC1	hypermethylated in cancer 1	2610	0	-1.47	0.186	Activated	<0.001
HMGB1	high mobility group box 1	3398	0	1.31	0.055	Activated	<0.001
HMGN5	high mobility group nucleosome binding domain 5	1012	0	1.18	0.722	Activated	0.043
IFI16	interferon, gamma-inducible protein 16	NA	NA	--	--	Activated	<0.001
IRF1	interferon regulatory factor 1	340	6E-92	29.63	<0.001	Activated	<0.001
IRF3	interferon regulatory factor 3	1828	0	-1.30	0.339	Activated	<0.001
IRF5	interferon regulatory factor 5	2565	0	-1.34	0.007	Activated	<0.001
IRF7	interferon regulatory factor 7	2003	0	2.14	<0.001	Activated	<0.001
LITAF	lipopolysaccharide-induced TNF factor	1755	0	1.50	<0.001	Activated	0.001
MYC	v-myc avian myelocytomatosis viral oncogene homolog	3086	0	-1.09	0.637	Activated	<0.001
MYCN	v-myc avian myelocytomatosis viral oncogene neuroblastoma derived homolog	NA	NA	--	--	Activated	<0.001
NFATC2	nuclear factor of activated T-cells, cytoplasmic, calcineurin-dependent 2	4740	0	-1.21	0.875	Activated	<0.001
NFE2L2	nuclear factor, erythroid 2-like 2	3191	0	1.84	<0.001	Activated	<0.001
NFkB (complex)	NFkB complex	NA	NA	--	--	Activated	<0.001
NLRC5	NLR family, CARD domain containing 5	5386	0	3.44	<0.001	Activated	0.002
NR0B1	nuclear receptor subfamily 0, group B, member 1	NA	NA	--	--	Activated	0.043

NRF1	nuclear respiratory factor 1	3779	0	1.18	0.594	Activated	0.006
REL	v-rel avian reticuloendotheliosis viral oncogene homolog	2773	0	-1.26	0.285	Activated	<0.001
RELA	v-rel avian reticuloendotheliosis viral oncogene homolog A	3180	0	1.30	0.026	Activated	<0.001
RELB	v-rel avian reticuloendotheliosis viral oncogene homolog B	1418	0	1.42	0.358	Activated	<0.001
SPDEF	SAM pointed domain containing ETS transcription factor	NA	NA	--	--	Activated	<0.001
STAT1	signal transducer and activator of transcription 1, 91kDa	4136	0	3.37	<0.001	Activated	<0.001
STAT2	signal transducer and activator of transcription 2, 113kDa	3216	0	2.11	<0.001	Activated	<0.001
STAT3	signal transducer and activator of transcription 3 (acute-phase response factor)	4268	0	1.78	<0.001	Activated	<0.001
STAT4	signal transducer and activator of transcription 4	3653	0	1.50	0.039	Activated	<0.001
TBX2	T-box 2	2087	0	-2.30	0.030	Activated	<0.001
TBX21	T-box 21	2953	0	2.99	<0.001	Activated	0.001
TFDP1	transcription factor Dp-1	2379	0	2.22	<0.001	Activated	<0.001
TLX1	T-cell leukemia homeobox 1	1862	0	-5.19	<0.001	Activated	0.007
XBP1	X-box binding protein 1	2116	0	2.29	<0.001	Activated	<0.001
BCL6	B-cell CLL/lymphoma 6	4955	0	1.23	0.270	Inhibited	<0.001
CDKN2A	cyclin-dependent kinase inhibitor 2A	425	2E-117	5.04	<0.001	Inhibited	<0.001
CNOT7	CCR4-NOT transcription complex, subunit 7	3593	0	-1.12	0.526	Inhibited	<0.001
FOXC2	forkhead box C2 (MFH-1, mesenchyme forkhead 1)	NA	NA	--	--	Inhibited	<0.001
HOXA9	homeobox A9	NA	NA	--	--	Inhibited	<0.001
HTT	huntingtin	15230	0	-1.07	0.718	Inhibited	<0.001
KDM5B	lysine (K)-specific demethylase 5B	8224	0	-1.41	0.002	Inhibited	<0.001
KLF4	Kruppel-like factor 4 (gut)	3077	0	-2.11	0.002	Inhibited	<0.001
LMX1B	LIM homeobox transcription factor 1, beta	NA	NA	--	--	Inhibited	0.002
MXI1	MAX interactor 1, dimerization protein	3094	0	-1.21	0.442	Inhibited	0.056
NEUROG3	neurogenin 3	NA	NA	--	--	Inhibited	0.368
NFIL3	nuclear factor, interleukin 3 regulated	2450	0	1.76	0.037	Inhibited	0.048
NKX2-3	NK2 homeobox 3	2684	0	-2.21	<0.001	Inhibited	<0.001
NOTCH4	notch 4	2819	0	-2.27	0.091	Inhibited	<0.001
NR1H2	nuclear receptor subfamily 1, group H, member 2	2489	0	1.31	0.718	Inhibited	<0.001
NR1H3	nuclear receptor subfamily 1, group H, member 3	2219	0	-1.05	0.761	Inhibited	<0.001
NUPR1	nuclear protein, transcriptional regulator, 1	666	0	12.73	<0.001	Inhibited	<0.001
Rb	Retinoblastoma group	NA	NA	--	--	Inhibited	<0.001
RB1	retinoblastoma 1	6334	0	1.28	0.078	Inhibited	<0.001
RBL1	retinoblastoma-like 1 (p107)	6156	0	2.29	<0.001	Inhibited	<0.001

RXRB	retinoid X receptor, beta	2498	0	-1.42	0.128	Inhibited	0.087
SIRT1	sirtuin 1	4370	0	-1.53	0.103	Inhibited	<0.001
SMAD6	SMAD family member 6	2461	0	-3.22	0.017	Inhibited	0.021
SMARCB1	SWI/SNF related, matrix associated, actin dependent regulator of chromatin, subfamily b, member 1	2295	0	1.20	0.469	Inhibited	<0.001
SNAI1	snail family zinc finger 1	2022	0	-1.85	0.195	Inhibited	<0.001
TAF4	TAF4 RNA polymerase II, TATA box binding protein (TBP)-associated factor, 135kDa	NA	NA	--	--	Inhibited	<0.001
TCF3	transcription factor 3	3712	0	-1.04	0.814	Inhibited	<0.001
THRB	thyroid hormone receptor, beta	1893	0	-1.85	0.015	Inhibited	<0.001
TOB1	transducer of ERBB2, 1	1680	0	-1.18	0.423	Inhibited	<0.001
TP53	tumor protein p53	1328	0	-1.10	0.598	Inhibited	<0.001
TRIM24	tripartite motif containing 24	4964	0	1.46	0.037	Inhibited	<0.001

B. TFs predicted to be activated/inhibited in infected splenic MΦ

Symbol	Entrez Gene Name	Blast Results		Differential Expression		IPA Prediction	
		Score	EValue	FC	FDR	Status	p-value
BRCA1	breast cancer 1, early onset	5633	0	1.10	0.923	Activated	0.011
CEBPA	CCAAT/enhancer binding protein (C/EBP), alpha	895	0	-1.11	0.851	Activated	<0.001
CEBPB	CCAAT/enhancer binding protein (C/EBP), beta	969	0	-1.33	0.542	Activated	<0.001
ELK1	ELK1, member of ETS oncogene family	4058	0	1.21	0.833	Activated	0.001
HAND2	heart and neural crest derivatives expressed 2	1633	0	--	--	Activated	0.008
HNF4A	hepatocyte nuclear factor 4, alpha	NA	NA	--	--	Activated	0.027
IFI16	interferon, gamma-inducible protein 16	NA	NA	--	--	Activated	0.001
IRF3	interferon regulatory factor 3	1828	0	-1.29	0.667	Activated	<0.001
IRF6	interferon regulatory factor 6	2691	0	-2.10	0.082	Activated	0.003
IRF7	interferon regulatory factor 7	2003	0	1.24	0.400	Activated	<0.001
JUNB/AP1	jun B proto-oncogene	2668	0	-1.50	0.369	Activated	<0.001
MEF2C	myocyte enhancer factor 2C	4462	0	-1.65	0.678	Activated	0.008
MYOCD	myocardin	53.6	1E-05	--	--	Activated	0.012
NFATC2	nuclear factor of activated T-cells, cytoplasmic, calcineurin-dependent 2	4740	0	--	--	Activated	<0.001
NFE2L2	nuclear factor, erythroid 2-like 2	3191	0	-1.19	0.732	Activated	<0.001
NFkB (complex)	NFkB complex	NA	NA	--	--	Activated	<0.001

NFKB1/p50	nuclear factor of kappa light polypeptide gene enhancer in B-cells 1	4715	0	-1.08	0.805	Activated	<0.001
POU2AF1	POU class 2 associating factor 1	1462	0	1.66	0.239	Activated	<0.001
RELA	v-rel avian reticuloendotheliosis viral oncogene homolog A	3180	0	-1.18	0.665	Activated	<0.001
SMARCB1	SWI/SNF related, matrix associated, actin dependent regulator of chromatin, subfamily b, member 1	2295	0	2.86	0.082	Activated	0.001
STAT1	signal transducer and activator of transcription 1, 91kDa	4136	0	2.99	<0.001	Activated	<0.001
TBX5	T-box 5	NA	NA	--	--	Activated	0.024
FOXA2	forkhead box A2	NA	NA	--	--	Inhibited	0.057
NKX2-3	NK2 homeobox 3	2684	0	-1.75	0.481	Inhibited	<0.001
Nr1h	Nr1h group	NA	NA	--	--	Inhibited	<0.001
NR1H3	nuclear receptor subfamily 1, group H, member 3	2219	0	-1.33	0.248	Inhibited	0.021
PIAS4	protein inhibitor of activated STAT, 4	2439	0	-1.45	0.275	Inhibited	<0.001
SMAD4	SMAD family member 4	4194	0	-1.00	0.993	Inhibited	<0.001
TRIM24	tripartite motif containing 24	4964	0	2.07	0.246	Inhibited	<0.001
TSC22D3	TSC22 domain family, member 3	2590	0	-1.01	0.988	Inhibited	<0.001
