# Supplementary material for: Transcriptional Profiling in Experimental Visceral Leishmaniasis Reveals a Broad Splenic Inflammatory Environment that Conditions Macrophages toward a Disease-Promoting Phenotype
Source: PLoS Pathog. 2017 Jan 31;13(1):e1006165. doi: 10.1371/journal.ppat.1006165 (PMC5283737; doi:10.1371/journal.ppat.1006165)
Supplement: S4 Table — (PDF) [file ppat.1006165.s009.pdf]

Table S4. M1- and M2-associated genes [1-27]

Gene Symbol	Entrez Gene Name	Blast Results		Spleen Tissue		Splenic MΦ[1]	
		Score	E Value	FC[2]	FDR[3]	FC	FDR
**M1-associated Genes**							
Ccl2	Chemokine (C-C motif) ligand 2	643	0	25.17	<0.001	1.36	0.577
Ccl3	Chemokine (C-C motif) ligand 3	623	4E-177	15.46	<0.001	3.28	<0.001
Ccl5	Chemokine (C-C motif) ligand 5	607	3E-172	16.30	<0.001	1.45	0.495
Cxcl10	Chemokine (C-X-C motif) ligand 10	618	2E-175	12.86	<0.001	1.28	0.365
Cxcl11	Chemokine (C-X-C motif) ligand 11	455	1E-126	104.37	<0.001	5.13	<0.001
Cxcl16	Chemokine (C-X-C motif) ligand 16	964	0	1.80	<0.001	1.08	0.839
Cxcl9	Chemokine (C-X-C motif) ligand 9	1067	0	29.35	<0.001	4.39	<0.001
Ido1	Indoleamine 2,3-dioxygenase 1	1274	0	368.73	<0.001	39.87	<0.001
Ifnγ	Interferon, gamma	706	0	52.15	<0.001	11.12	<0.001
Il6	Interleukin 6 (interferon, beta 2)	1074	0	-	-	5.04	0.007
Irf1	Interferon regulatory factor 1	340	6E-92	29.63	<0.001	-1.08	0.947
Irf7	Interferon regulatory factor 7	2003	0	2.14	<0.001	1.24	0.400
Irg1	Immunoresponsive 1 homolog (mouse)	2107	0	365.35	<0.001	7.77	<0.001
Slamf7	SLAM family member 7	769	0	2.86	<0.001	1.91	<0.001
Socs3	Suppressor of cytokine signaling 3	1799	0	4.42	<0.001	1.30	0.393
Stat1	Signal transducer and activator of transcription 1, 91kDa	4136	0	3.37	<0.001	2.99	<0.001
Fcgr1a	Fc receptor, IgG, high affinity I	1577	0	1.37	0.013	1.93	<0.001
Tnf	Tumor necrosis factor	1142	0	1.16	0.707	-1.43	0.400
Ccr7	Chemokine (C-C motif) receptor 7	1743	0	-2.43	<0.001	-1.15	0.703
Il1b	Interleukin 1, beta	1294	0	-2.09	<0.001	1.95	<0.001
**M2-associated Genes**							
Arg1	Arginase 1	1498	0	35.71	<0.001	5.85	<0.001
Ccl11	Chemokine (C-C motif) ligand 11	823	0	20.5	<0.001	-	-
Ccl17	Chemokine (C-C motif) ligand 17	383	4E-105	70.91	<0.001	4.53	0.009
Ccl2	Chemokine (C-C motif) ligand 2	643	0	25.17	<0.001	1.36	0.577
Ccl22	Chemokine (C-C motif) ligand 22	1023	0	4.01	<0.001	1.26	0.743
Cd226	CD226 molecule	1948	0	3.01	0.005	1.41	0.593
Cebpb	CCAAT/enhancer binding protein (C/EBP), beta	969	0	3.73	<0.001	-1.33	0.542
Chi3l1	Chitinase 3-like 1 (cartilage glycoprotein-39)	1674	0	20.15	<0.001	3.78	0.044
Cxcl16	Chemokine (C-X-C motif) ligand 16	964	0	1.80	<0.001	1.08	0.839
F13a1	Coagulation factor XIII, A1 polypeptide	4601	0	1.82	<0.001	1.61	0.410
Fn1	Fibronectin 1	11400	0	-1.7	0.146	1.1	0.925
Socs1	Suppressor of cytokine signaling 1	1701	0	9.89	<0.001	1.17	0.772
Stat3	Signal transducer and activator of transcription 3	4268	0	1.78	<0.001	-1.08	0.889
Il1rn	Interleukin 1 receptor antagonist	1557	0	19.65	<0.001	1.60	0.072
Socs2	Suppressor of cytokine signaling 2	2493	0	-2.56	<0.001	3.65	<0.001
Stab1	Stabilin 1	9499	0	-1.80	0.001	-2.08	0.198
Klf4	Kruppel-like factor 4 (gut)	3077	0	-2.11	0.002	-1.47	0.436
Msr1	Macrophage scavenger receptor 1	1570	0	-1.62	<0.001	-1.46	0.065
Pparg	Peroxisome Proliferator-Activated Receptor Gamma	2791	0	-1.3	0.376	-1.6	0.101
Cd36	CD36 molecule (thrombospondin receptor)	2486	0	-3.62	<0.001	1.02	0.977
Cd93	CD93 molecule	2578	0	-1.90	<0.001	-1.45	0.520
Cd163	CD163 molecule	2477	0	-5.27	0.035	-2.20	0.209
Cd209a	CD209a antigen	1169	0	-7.08	<0.001	1.41	0.635
Ccr2	Chemokine (C-C motif) receptor 2	1296	0	1.14	0.638	2.70	0.063
Akt1	v-akt murine thymoma viral oncogene homolog 1	3110	0	-1.09	0.514	-1.69	0.056
Fcer1g	Fc fragment of IgE, high affinity I, receptor for; gamma polypeptide	598	2E-169	-1.42	0.006	-1.38	0.385
Igf1	Insulin-like growth factor 1 (somatomedin C)	4298	0	-5.24	<0.001	-1.13	0.933
Il10	Interleukin 10	1397	0	-1.27	0.611	1.51	0.051
Il27ra	Interleukin 27 receptor, alpha	2053	0	1.06	0.849	-1.52	0.538

[1] Macrophage
[2] Fold-change (infected/uninfected)
[3] False Discovery Rate

References

1. Murray PJ, Wynn TA (2011) Protective and pathogenic functions of macrophage subsets. Nat Rev Immunol 11: 723-737.
2. Biswas SK, Mantovani A (2010) Macrophage plasticity and interaction with lymphocyte subsets: cancer as a paradigm. Nat Immunol 11: 889-896.
3. Martinez FO (2009) The transcriptome of human monocyte subsets begins to emerge. J Biol 8: 99.

4. Martinez FO (2011) Regulators of macrophage activation. Eur J Immunol 41: 1531-1534.
5. Martinez FO (2012) Analysis of gene expression and gene silencing in human macrophages. Curr Protoc Immunol Chapter 14: Unit 14.28.11-23.
6. Martinez FO, Gordon S (2014) The M1 and M2 paradigm of macrophage activation: time for reassessment. F1000Prime Rep 6: 13.
7. Martinez FO, Gordon S, Locati M, Mantovani A (2006) Transcriptional profiling of the human monocyte-to-macrophage differentiation and polarization: new molecules and patterns of gene expression. J Immunol 177: 7303-7311.
8. Martinez FO, Helming L, Milde R, Varin A, Melgert BN, et al. (2013) Genetic programs expressed in resting and IL-4 alternatively activated mouse and human macrophages: similarities and differences. Blood 121: e57-69.
9. Martinez FO, Sica A, Mantovani A, Locati M (2008) Macrophage activation and polarization. Front Biosci 13: 453-461.
10. van de Garde MD, Martinez FO, Melgert BN, Hylkema MN, Jonkers RE, et al. (2014) Chronic exposure to glucocorticoids shapes gene expression and modulates innate and adaptive activation pathways in macrophages with distinct changes in leukocyte attraction. J Immunol 192: 1196-1208.
11. Mosser DM, Edwards JP (2008) Exploring the full spectrum of macrophage activation. Nat Rev Immunol 8: 958-969.
12. Ohteki T (2002) Critical role for IL-15 in innate immunity. Curr Mol Med 2: 371-380.
13. Wang XF, Wang HS, Wang H, Zhang F, Wang KF, et al. (2014) The role of indoleamine 2,3-dioxygenase (IDO) in immune tolerance: Focus on macrophage polarization of THP-1 cells. Cell Immunol 289: 42-48.
14. Mantovani A, Sica A, Locati M (2005) Macrophage polarization comes of age. Immunity 23: 344-346.
15. Sica A, Mantovani A (2012) Macrophage plasticity and polarization: in vivo veritas. J Clin Invest 122: 787-795.
16. Ambarus CA, Krausz S, van Eijk M, Hamann J, Radstake TR, et al. (2012) Systematic validation of specific phenotypic markers for in vitro polarized human macrophages. J Immunol Methods 375: 196-206.
17. Porta C, Rimoldi M, Raes G, Brys L, Ghezzi P, et al. (2009) Tolerance and M2 (alternative) macrophage polarization are related processes orchestrated by p50 nuclear factor kappaB. Proc Natl Acad Sci U S A 106: 14978-14983.
18. Garzetti L, Menon R, Finardi A, Bergami A, Sica A, et al. (2013) Activated macrophages release microvesicles containing polarized M1 or M2 mRNAs. J Leukoc Biol.
19. Tiemessen MM, Jagger AL, Evans HG, van Herwijnen MJ, John S, et al. (2007) CD4+CD25+Foxp3+ regulatory T cells induce alternative activation of human monocytes/macrophages. Proc Natl Acad Sci U S A 104: 19446-19451.
20. Huber S, Hoffmann R, Muskens F, Voehringer D (2010) Alternatively activated macrophages inhibit T-cell proliferation by Stat6-dependent expression of PD-L2. Blood 116: 3311-3320.
21. Stahl M, Schupp J, Jager B, Schmid M, Zissel G, et al. (2013) Lung collagens perpetuate pulmonary fibrosis via CD204 and M2 macrophage activation. PLoS One 8: e81382.
22. Sica A, Schioppa T, Mantovani A, Allavena P (2006) Tumour-associated macrophages are a distinct M2 polarised population promoting tumour progression: potential targets of anti-cancer therapy. Eur J Cancer 42: 717-727.
23. Kurowska-Stolarska M, Stolarski B, Kewin P, Murphy G, Corrigan CJ, et al. (2009) IL-33 amplifies the polarization of alternatively activated macrophages that contribute to airway inflammation. J Immunol 183: 6469-6477.
24. Van den Bossche J, Bogaert P, van Hengel J, Guerin CJ, Berx G, et al. (2009) Alternatively activated macrophages engage in homotypic and heterotypic interactions through IL-4 and polyamine-induced E-cadherin/catenin complexes. Blood 114: 4664-4674.
25. Puig-Kroger A, Sierra-Filardi E, Dominguez-Soto A, Samaniego R, Corcuera MT, et al. (2009) Folate receptor beta is expressed by tumor-associated macrophages and constitutes a marker for M2 anti-inflammatory/regulatory macrophages. Cancer Res 69: 9395-9403.
26. Patil V, Zhao Y, Shah S, Fox BA, Rommereim LM, et al. (2014) Co-existence of classical and alternative activation programs in macrophages responding to Toxoplasma gondii. Int J Parasitol 44: 161-164.
27. Spence S, Fitzsimons A, Boyd CR, Kessler J, Fitzgerald D, et al. (2013) Suppressors of cytokine signaling 2 and 3 diametrically control macrophage polarization. Immunity 38: 66-78.
