# Supplementary material for: Transcriptional Profiling in Experimental Visceral Leishmaniasis Reveals a Broad Splenic Inflammatory Environment that Conditions Macrophages toward a Disease-Promoting Phenotype
Source: PLoS Pathog. 2017 Jan 31;13(1):e1006165. doi: 10.1371/journal.ppat.1006165 (PMC5283737; doi:10.1371/journal.ppat.1006165)
Supplement: S5 Table — (PDF) [file ppat.1006165.s010.pdf]

Table S5. Hamster Interferon Response Genes *

Gene Category	Gene Symbol	Description	Spleen Infection		Splenic MΦ Infection	
			FC	FDR	FC	FDR
IFI Family	IFIH1	interferon induced with helicase C domain 1	-1.4	0.4	-1.4	0.4
	IFI6	interferon, alpha-inducible protein 6	NF	NF	NF	NF
	IFI16	interferon, gamma-inducible protein 16	NF	NF	NF	NF
	IFIT5	interferon-induced protein with tetratricopeptide repeats 5	NF	NF	NF	NF
	IFI44	interferon-induced protein 44	-1.2	0.1	1	1
	IFIT3	interferon-induced protein with tetratricopeptide repeats 3	2.1	<0.001	1.5	0.6
	IFI44L	interferon-induced protein 44-like	-1.2	0.1	1.9	<0.001
	IFIT2	interferon-induced protein with tetratricopeptide repeats 2	2.8	<0.001	2.1	0.15
	IFITM3	interferon induced transmembrane protein 3	1.1	0.42	-1.5	0.55
	IFI35	interferon-induced protein 35	1.4	0.012	-1.5	0.57
	IFITM1	interferon induced transmembrane protein 1	20.5	<0.001	-	-
	IFI203	interferon activated gene 203	3	<0.001	2.2	0.05
	IFI204	interferon activated gene 204	1.8	<0.001	2.9	<0.001
	IFI205	interferon activated gene 205	1.8	<0.001	2.9	<0.001
	IFI27L2B	interferon, alpha-inducible protein 27 like 2B	88.2	<0.001	-	-
	IFIT1	interferon-induced protein with tetratricopeptide repeats 1	7.8	<0.001	-1	0.932
	IFIT1LB	interferon-induced protein with tetratricopeptide repeats 1B-like	1.5	0.296	-	-
	IFI27	interferon, alpha-inducible protein 27	1	0.949	-1	0.992
	IFI47	interferon gamma inducible protein 47	4.8	<0.001	3.7	<0.001
	MNDA	myeloid cell nuclear differentiation antigen	1.8	<0.001	1.5	<0.001
Interferon stimulated genes	ISG20	interferon stimulated exonuclease gene 20kDa	1.6	0.225	2.1	0.202
	ISG15	ISG15 ubiquitin-like modifier	3.8	<0.001	2.1	0.143
Antiviral effectors	OAS1G	2'-5' oligoadenylate synthetase 1G	1.2	0.856	-	-
	OAS1K	2 ' -5 ' oligoadenylate synthetase 1K	3.2	<0.001	11.4	<0.001
	OAS3	2'-5'-oligoadenylate synthetase 3, 100kDa	3.9	<0.001	-	-
	OAS2	2'-5'-oligoadenylate synthetase 2, 69/71kDa	-	-	2.1	0.381
	OASL	2'-5'-oligoadenylate synthetase-like	5.9	<0.001	-1	0.9
	MX1	MX dynamin-like GTPase 1	1.1	0.798	1.7	0.15
	MX2	MX dynamin-like GTPase 2	-30.7	<0.001	-	-
	DDX58	DEAD (Asp-Glu-Ala-Asp) box polypeptide 58	1.5	<0.001	1.4	0.409
	MAVS	mitochondrial antiviral signaling protein	1.3	0.5	-1.1	0.934
Signal Transducer and Activator of Transcription (STAT)	STAT1	signal transducer and activator of transcription 1, 91kDa	3.4	<0.001	3	<0.001
	STAT2	signal transducer and activator of transcription 2, 113kDa	2.1	<0.001	1.1	0.936
Interferon regulatory factors	IRF1	interferon regulatory factor 1	3.2	<0.001	-1.1	0.679
	IRF2	interferon regulatory factor 2	1.1	0.778	-1	0.993
	IRF3	interferon regulatory factor 3	-1.3	0.339	-1.3	0.667
	IRF4	interferon regulatory factor 4	2	<0.001	1	0.956
	IRF7	interferon regulatory factor 7	2.1	<0.001	1.2	0.4
	IRF8	interferon regulatory factor 8	1.4	<0.001	-1.7	0.01
Cytokines / Chemokines	CXCL5	chemokine (C-X-C motif) ligand 5	94.9	<0.001	2.7	0.188
	CXCL9	chemokine (C-X-C motif) ligand 9	29.4	<0.001	4.4	<0.001
	CXCL10	chemokine (C-X-C motif) ligand 10	12.9	<0.001	1.3	0.365
	CXCL11	chemokine (C-X-C motif) ligand 11	-1.1	0.657	1.2	0.796
	CCL8	chemokine (C-C motif) ligand 8	120.5	<0.001	17.2	<0.001
	CCR3	chemokine (C-C motif) receptor 3	-4	<0.001	1.5	0.04
	IFNG	interferon, gamma	52.2	<0.001	11.1	<0.001
	IFNGR1	interferon gamma receptor 1	-1.4	0.004	1.3	0.435
	IL15RA	interleukin 15 receptor, alpha	8.1	<0.001	3.1	<0.001
	SPP1	secreted phosphoprotein 1	54.8	<0.001	5.9	<0.001
	TNFSF10	tumor necrosis factor (ligand) superfamily, member 10	1.9	<0.001	5.2	<0.001
	TNFSF13B	tumor necrosis factor (ligand) superfamily, member 13b	2	<0.001	2.1	0.253
Tripartite motif (TRIM) protein family	TRIM5	tripartite motif containing 5	-1.1	0.761	-1.1	0.917
	TRIM10	tripartite motif containing 10	5.7	<0.001	-	-
	TRIM12A	tripartite motif-containing 12A	-1.5	0.204	2	0.327
	TRIM26	tripartite motif containing 26	1.1	0.785	-1.1	0.928
	TRIM30A	tripartite motif-containing 30A	1.6	<0.001	1.4	0.422
	TRIM56	tripartite motif containing 56	1.2	0.552	-1.1	0.927
	TRIM59	tripartite motif containing 59	1.6	0.001	1.2	0.742
	TRIM58	tripartite motif containing 58	3.7	0.008	-	-
	TRIM65	tripartite motif containing 65	-1.4	0.446	-	-

	TRIM67	tripartite motif containing 67	-2.2	0.039	-	-
	TRIM72	tripartite motif containing 72, E3 ubiquitin protein ligase	-	-	2.4	0.358
Guanylate-Binding Proteins	GBP1	guanylate binding protein 1, interferon-inducible	187.6	<0.001	18.1	<0.001
	GBP2	guanylate binding protein 2, interferon-inducible	11.2	<0.001	6.1	<0.001
	GBP2B	guanylate binding protein 2b	7.4	<0.001	8.6	0.25
	GBP3	guanylate binding protein 3	1.7	0.003	2	0.025
	GBP4	guanylate binding protein 4	1.7	0.003	2	0.025
	GBP5	guanylate binding protein 5	6.6	<0.001	5.5	<0.001
	GBP6	guanylate binding protein family, member 6	1.7	0.003	2	0.025
	GBP7	guanylate binding protein 7	3.5	<0.001	2.2	0.201
	GBP8	guanylate-binding protein 8	10.1	<0.001	8.2	<0.001
	GBP9	guanylate-binding protein 9	1.7	0.003	2	0.025
	GBP10	guanylate-binding protein 10	1.7	0.003	2	0.025
	GBP11	guanylate binding protein 11	1.7	0.003	2	0.025
T cell related	CD274	CD274 molecule (PDCD1LG1, programmed cell death 1 ligand 1)	2.6	<0.001	1.9	<0.001
	LAG3	lymphocyte-activation gene 3	27.3	<0.001	2.1	0.286
	LILRA6	leukocyte immunoglobulin-like receptor, subfamily A (with TM domain), member 6	22.7	<0.001	2.7	0.002
	PDCD1LG2	programmed cell death 1 ligand 2	26.9	<0.001	3.9	<0.001
Miscellaneous	TAP1	transporter 1, ATP-binding cassette, sub-family B (MDR/TAP)	2.7	<0.001	2.2	<0.001
	TAP2	transporter 2, ATP-binding cassette, sub-family B (MDR/TAP)	2.3	<0.001	1.3	0.577
	SOCS1	suppressor of cytokine signaling 1	5.3	0.001	-1	0.979
	SOCS2	suppressor of cytokine signaling 2	-2.6	<0.001	3.6	<0.001
	SOCS3	suppressor of cytokine signaling 3	4.4	<0.001	1.3	0.393
	IDO1	indoleamine 2,3-dioxygenase 1	368.7	<0.001	39.9	<0.001
	IRG1	immunoresponsive 1 homolog (mouse)	365.3	<0.001	7.8	<0.001
	WARS	tryptophanyl-tRNA synthetase	8.4	<0.001	3.8	<0.001
	TLR3	toll-like receptor 3	-2.6	0.046	-	-
	TLR9	toll-like receptor 9	-2.2	<0.001	-1.6	0.259
	ANKRD22	ankyrin repeat domain 22	NF	NF	NF	NF
	CMPK2	cytidine monophosphate (UMP-CMP) kinase 2, mitochondrial	2	<0.001	1.6	0.2
	DTX3L	deltex 3 like, E3 ubiquitin ligase	1.7	0.005	2.3	0.086
	EPSTI1	epithelial stromal interaction 1 (breast)	2.5	<0.001	2.5	0.022
	HERC6	HECT and RLD domain containing E3 ubiquitin protein ligase family member 6	2.1	<0.001	1.9	0.065
	LAP3	leucine aminopeptidase 3	2.5	<0.001	-1	0.989
	LY6E	lymphocyte antigen 6 complex, locus E	1.4	0.002	1.8	0.081
	PARP9	poly (ADP-ribose) polymerase family, member 9	2	<0.001	3.6	<0.001
	PARP12	poly (ADP-ribose) polymerase family, member 12	1.7	<0.001	1.8	0.031
	PGAP1	post-GPI attachment to proteins 1	-1.4	0.158	1	0.994
	PML	promyelocytic leukemia	1.7	<0.001	2.2	0.028
	RGL1	ral guanine nucleotide dissociation stimulator-like 1	-1.5	<0.001	1.6	0.007
	RSAD2	radical S-adenosyl methionine domain containing 2	6	<0.001	2.4	<0.001
	SAMD9L	sterile alpha motif domain containing 9-like	1.7	<0.001	2.2	<0.001
	USP18	ubiquitin specific peptidase 18	2.3	<0.001	1	0.957
	XAF1	XIAP associated factor 1	1.6	0.004	2.5	0.001
	APOL6	apolipoprotein L, 6	11.7	<0.001	29.8	<0.001
	APOL2	apolipoprotein L, 2	9.6	<0.001	-	-
	APOBEC3	apolipoprotein B mRNA editing enzyme, catalytic polypeptide 3	1.6	<0.001	1.9	0.079
	ARHGEF3	Rho guanine nucleotide exchange factor (GEF) 3	2.4	<0.001	3.3	0.003
	ARHGEF10	Rho guanine nucleotide exchange factor (GEF) 10	-1.9	0.016	-1.5	0.511
	BATF	basic leucine zipper transcription factor, ATF-like	6.3	<0.001	2	0.228
	CD163	CD163 molecule	-5.3	0.035	-2.2	0.208
	CD38	CD38 molecule	3.9	<0.001	1.9	<0.001
	CHRNA6	cholinergic receptor, nicotinic, alpha 6 (neuronal)	-3.5	<0.001	-8.6	<0.001
	EHD4	EH-domain containing 4	-1.2	0.169	1.1	0.737
	EPHB2	EPH receptor B2	-1	0.978	-	-
	FASLG	Fas ligand (TNF superfamily, member 6)	3.4	<0.001	-	-
	GNB4	guanine nucleotide binding protein (G protein), beta polypeptide 4	-1.8	<0.001	-1	0.968
	LACTB	lactamase, beta	-1.5	0.001	1.1	0.871
	LMNB1	lamin B1	3.2	<0.001	1.6	0.116
	MB21D1	Mab-21 domain containing 1	3	<0.001	-1.1	0.935
	MEFV	Mediterranean fever	1.9	0.006	1.5	0.149
	MLKL	mixed lineage kinase domain-like	1.6	0.002	1.7	0.052
	MS4A6A	membrane-spanning 4-domains, subfamily A, member 6A	3.2	<0.001	9.9	<0.001
	MSR1	macrophage scavenger receptor 1	-1.5	0.022	-2	0.033
	MYOF	myoferlin	11	<0.001	7.4	<0.001
	NCOA7	nuclear receptor coactivator 7	2.5	0.003	1.6	0.262
	NEMF	nuclear export mediator factor	1.1	0.796	-1.2	0.796

	PI4K2B	Phosphatidylinositol 4-kinase type 2 beta; Phosphatidylinositol 4-kinase type 2-beta	1.2	0.634	1.2	0.841
	PLSCR1	phospholipid scramblase 1	2	<0.001	1.8	0.064
	PRLR	prolactin receptor	4.4	0.029	-	-
	RARG	retinoic acid receptor, gamma	-1.7	0.42	-	-
	RASGRP3	RAS guanyl releasing protein 3 (calcium and DAG-regulated)	2.1	0.019	-	-
	RGL1	ral guanine nucleotide dissociation stimulator-like 1	-1.5	<0.001	1.6	0.007
	RTP4	receptor (chemosensory) transporter protein 4	1.8	<0.001	1.5	0.535
	SAMD9L	sterile alpha motif domain containing 9-like	1.7	<0.001	2.2	<0.001
	SAT1	spermidine/spermine N1-acetyltransferase 1	2.4	<0.001	-1.2	0.6
	SCUBE2	signal peptide, CUB domain, EGF-like 2	-2.4	<0.001	-1.5	0.617
	SERPING1	serpin peptidase inhibitor, clade G (C1 inhibitor), member 1	2.5	<0.001	4.5	0.002
	SLC11A1	solute carrier family 11 (proton-coupled divalent metal ion transporter), member 1	2.4	<0.001	1.4	0.404
	SP100	SP100 nuclear antigen	1.7	0.11	1.6	0.465
	SP110	SP110 nuclear body protein	1.5	<0.001	1.7	0.014
	SP140	SP140 nuclear body protein	1.8	0.406	-	-
	TMEM140	transmembrane protein 140	2	<0.001	1	0.977
	TRAFD1	TRAF-type zinc finger domain containing 1	1.6	<0.001	1.5	0.12
	TRPM2	transient receptor potential cation channel, subfamily M, member 2	3.8	<0.001	5	<0.001
	UBE2L6	ubiquitin-conjugating enzyme E2L 6	2.7	<0.001	2.1	0.217
	ZBP1	Z-DNA binding protein 1	2.4	<0.001	1.9	0.372
	ZNFX1	zinc finger, NFX1-type containing 1	1.4	0.163	1	1

* IFN-response reference geneset manually curated from:

1. Ferreira RC, Guo H, Coulson RM, Smyth DJ, Pekalski ML, et al. (2014) A type I interferon transcriptional signature precedes autoimmunity in children genetically at risk for type 1 diabetes. Diabetes 63: 2538-2550.
2. Liu SY, Sanchez DJ, Aliyari R, Lu S, Cheng G (2012) Systematic identification of type I and type II interferon-induced antiviral factors. Proc Natl Acad Sci U S A 109: 4239-4244.
3. Waddell SJ, Popper SJ, Rubins KH, Griffiths MJ, Brown PO, et al. (2010) Dissecting interferon-induced transcriptional programs in human peripheral blood cells. PLoS One 5: e9753.
