# Supplementary material for: Transcriptional Profiling in Experimental Visceral Leishmaniasis Reveals a Broad Splenic Inflammatory Environment that Conditions Macrophages toward a Disease-Promoting Phenotype
Source: PLoS Pathog. 2017 Jan 31;13(1):e1006165. doi: 10.1371/journal.ppat.1006165 (PMC5283737; doi:10.1371/journal.ppat.1006165)
Supplement: S6 Table — (PDF) [file ppat.1006165.s011.pdf]

Table S6. Primer sequences used for real time RT-PCR assays

Hamster Target Gene	Primers Sequences (5’-3’)
CCL17	F - GTGCTGCCTGGAGATCTTCA<br>R - TGGCATCCCTGGGACACT
Arginase 1	F - ACCTATGTGTCATTTGGGTGGA<br>R - GCAGATATGCAGGGAGTCACC
Chi3L1	F - CTGCTCCAGTGCTGCTCTAC<br>R - AAGCAGCTTCCATCACCTTC
SOCS1	F - TTCCTGGAACCACGTGGGA<br>R - CATGGAGAGGTAGGAGTGGAG
IDO	F -CACATGTCTCCCACTGAAGG<br>R - CAGGCACTGAATGTCTGAGG
IRG1	F - GAGAGGGTTGTGCTCAGGAT<br>R - CCACGTACTGGAAGGAGTGA
IFNγ	F - AATATCTTGACGAACTGGCAAA<br>R - CCTTCAAGGCTTCAAAGAGTTT
CXCL9	F - TGGGTATCATCCTCCTGGAC<br>R - AATGAGGACCTGGAGCAAAC
CXCL10	F - TGGAAATTATTCCTGCAAGTCA<br>R - GTGATCGGCTTCTCTCTGGT
iNOS	F - TGAGCCACTGAGTTCTCCTAAGG<br>R - TCCTATTTCAACTCCAAGATGTTCTG
CCL11	F- CTATCCCAGTTTCCTGCTGC<br>R- GGTCAGCACAGATATCCTTGC
IL-10	F- TAAGGGTTACTTGGGTTGCC<br>R- CAAGGCTGTGGAACAGGTGAA
18s	F - ACCGCAGCTAGGAATAATGGA<br>R - GCCTCAGTTCCGAAAACCA
